# Electroanalytical Methods to Establish the Role of Buffer and Electrolyte Components in Water Denitrification Using a Copper-Based Bioinspired Electrocatalyst

**DOI:** 10.1021/acsmeasuresciau.5c00203

**Published:** 2026-02-10

**Authors:** Vanessa A. Hulse, Katy A. Knecht, Frank R. Fronczek, Noémie Elgrishi

**Affiliations:** Department of Chemistry, 5779 Louisiana State University, 232 Choppin Hall, Baton Rouge, Louisiana 70803, United States

**Keywords:** electroanalytical, water denitrification, oxyanion, nitrite electroreduction, copper, Me_6_Tren, electrocatalysis

## Abstract

The overuse of fertilizers in modern agricultural practices has contributed to the accumulation of nitrogen oxyanions in water systems. These nitrogen oxyanions, including nitrite and nitrate, pose risks to human and marine health. Of these contaminants, nitrite is especially problematic for aquatic life. Chemical methods for reducing nitrite in water are limited by cost and by the risk of secondary contamination. The electrochemical reduction of nitrite is a promising method to solve these issues. Here we report the electrocatalytic reduction of nitrite to nitric oxide in quantitative Faradaic yields with a copper-based electrocatalyst. Electroanalytical methods enable the discussion of mechanistic details, in particular with respect to the catalyst resting state as well as the unexpected role of the buffer and other electrolyte components on electrochemical activity. This work emphasizes the noninnocence of buffers and other electrolyte components, the choice of which should be of critical importance when evaluating the electrochemical activity of new homogeneous electrocatalysts.

## Graphical abstract

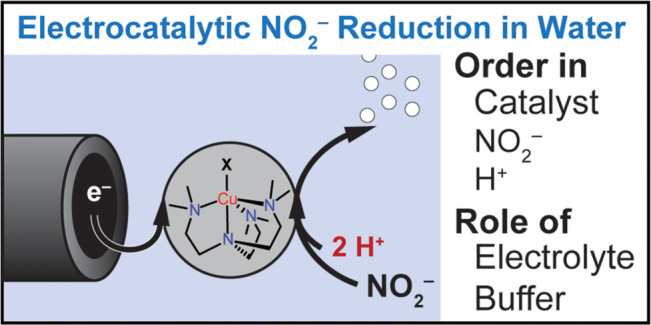

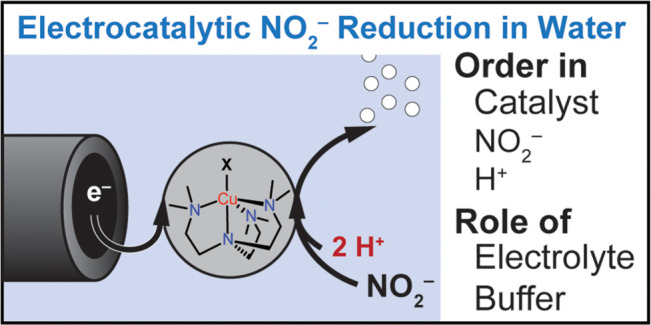

## Introduction

Oxyanion pollution is emerging as an increasing issue. Of these, oxyanions from the nitrogen cycle are garnering increasing interest due to their deleterious impact on health and the environment, as well as their relevance to agriculture and energy storage. Due to overfertilization, both nitrate and nitrite have been found in increasing amounts in water systems.^1^ Nitrite, in particular, is acutely toxic to many species of fish^2,3^ and can contribute to eutrophication.^4^ Previous reports on the removal of nitrite from water have included adsorption,^5,6^ ion-exchange,^7,8^ microbial treatment,^9^ reverse osmosis,^10^ and electrodialysis.^10^ Electrochemical reduction presents advantages over these methods, such as lowering costs, energy requirements, and avoiding the formation of secondary contaminants.^11,12^ Some reports have demonstrated systems able to perform the electrocatalytic reduction of nitrate.^13,14^ The development of molecular electrocatalysts for the reduction of N_2_O has also been an area of growing interest, with reports on copper, iron, and rhenium-based systems, in some cases producing N_2_, in organic solvents.^15–19^ More work has been reported on the electrochemical reduction of nitrite. In general, cobalt and iron-centered catalysts have been reported to reduce nitrite to ammonia/ammonium, which remain in solution.^20–24^ Another pathway produces gases, effectively removing nitrogen-containing products from water. These typically rely on copper catalysts.^25–27^ Work in organic solvents has demonstrated the impact of including a pendant acid arm on catalysis,^28^ as well as the production of both NO and N_2_O in organic solvents^29^ and in water,^30^ the latter is the focus of the current work. Critically, previous reports emphasized the difficulty of identifying appropriate catalysts or catalytic media, with reports of unexpected lack of activity or of activity switches depending on experimental conditions.^20,31^ In this work, we elucidate the intricate equilibria between buffer, electrolyte, and catalyst components that give rise to electrocatalytic nitrite reduction in water. The electroanalytical methods described can serve as a template for tackling future challenging systems and help rationalize and predict the activity of nitrite electroreduction catalysts.

The system chosen (Scheme 1) for this study features a copper center in a nitrogen-rich ligand field. Similar to the enzymatic system, nitrite reductase,^32^ it is expected to electrocatalytically reduce nitrite to NO in water, effectively removing nitrogen-containing species from solution.

**Scheme 1:**
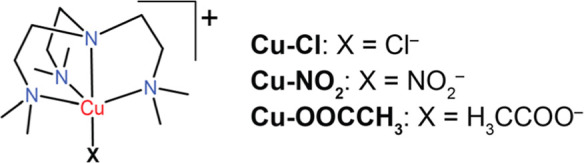
Structure of CuMe_6_Tren–X, where X = Cl^–^, NO_2_^–^, H_3_CCOO^–^ Abbreviated as **Cu–Cl**, **Cu–NO**_**2**_, and **Cu–OOCCH**_**3**_

## Experimental Section

### Materials and Reagents

The following chemicals were used as received from commercial sources: copper­(II) trifluoromethanesulfonate (BeanTown Chemical, 98%), copper­(II) chloride (Ward’s Science, lab grade), copper­(II) acetate monohydrate (Sigma-Aldrich, ≥98%), tris­(2-dimethylaminoethyl)­amine (Me_6_Tren, Alfa Aesar, ≥99%), cobalt­(II) tetraphenylporphyrin (Strem Chemicals, 99.3%), sodium nitrite for ion-exchange chromatography calibration (BeanTown chemical, ≥99.999%), sodium nitrate for ion-exchange chromatography calibration (Acros Organics, 99.999%), acetonitrile (Honeywell, ≥99.9%), pentane (BDH Chemicals, ≥98.0%), 2-propanol (JT Baker, ≥99.9%), dichloromethane (BDH Chemicals, ACS grade), methanol (EMD Millipore, ACS grade), ethanol (Sigma-Aldrich, ≥99.5%), hexane (BDH, ACS grade), and diethyl ether (EMD Millipore, ACS grade). Acetonitrile and methanol for mass spectrometry were from MilliporeSigma, LC–MS grade. Ultrapure Millipore deionized water was obtained using a Milli-Q Advantage A10 Direct water purification system, with a resistivity of 18.2 MΩ cm at 25.0 °C.

The following commercial chemicals were purified before use: potassium chloride was recrystallized by diffusion of ethanol into water, potassium nitrate was recrystallized from hot water, and sodium nitrite was dried under vacuum and stored in an inert atmosphere glovebox.

### Instrumentation

All pH measurements were made using a Mettler Toledo InLab MicroPro-ISM pH probe. Before each use, the pH probe was calibrated with pH buffers 4.01, 7.00, and 10.01 (Mettler Toledo).

Electrochemical measurements were made using a Biologic SP-300 potentiostat. Cyclic voltammetry experiments used a three-electrode cell with a 3.00 mm glassy carbon working electrode (CH Instruments), a 2 mm platinum counter electrode (CH Instruments), and a Ag/AgCl in 1.00 M KCl reference electrode stored in 1.00 M KCl in water and rinsed before use. Electrodes were polished with 0.05 μm alumina powder (Electron Microscopy Services) on a microcloth polishing pad (CH Instruments). To remove any trace alumina powder after polishing, each electrode was rinsed with water and sonicated before rinsing and drying under N_2_. Cyclic voltammograms (CVs) were plotted from data collected with 10.0 mL of solution in a borosilicate scintillation vial with the electrodes fed through a custom-made PTFE cap. Solutions were sparged with N_2_ for at least 20 min before collecting scans and a stream of N_2_ was blanketing the solution during scans. All controlled potential electrolysis (CPE) experiments used a two-compartment bulk electrolysis cell with either a glassy carbon or a 100 PPI reticulated vitreous carbon (RVC, Duocel) working electrode, a platinum wire counter electrode, and a Ag/AgCl reference electrode. The counter electrode compartment was separated from the main compartment with a 4–5.5 μm frit. The counter electrode compartment held 5.00 mL, and the working electrode compartment held 10.0 mL of solution. The counter electrode compartment solution was sparged with N_2_ for at least 10 min and the working electrode compartment solution was sparged with N_2_ for at least 20 min before data collection. N_2_ was left blanketing the entire cell during data collection. All electrochemical data is plotted using the IUPAC convention.

Ion-exchange chromatography data were collected with a Metrohm 930 Compact IC Flex instrument equipped with a conductivity detector at 40 °C. The eluent used was a 3.6 mM Na_2_CO_3_ solution at a flow rate of 0.7 mL/min and the column was a Metrosep A Supp 7—250/4.0 at 45 °C. All samples were diluted 100-fold before injection into the 20 μL injection loop. The experimental concentrations of nitrite and nitrate were determined using 6-point calibration curves obtained with standard solutions.

UV–vis measurements were collected on an Ocean Optics DH-2000-BAL UV–vis–NIR light source coupled with optic fibers to an Ocean FX spectrometer detector or an Agilent Cary 5000 UV–vis–NIR spectrophotometer with a 1 cm quartz cuvette. Weighted adjacent averaging over 1 nm was used when necessary. High-resolution mass spectrometry analysis was performed in the LSU Chemistry Mass Spectrometry Facility, on instruments as specified throughout. FTIR spectra were collected using a Bruker Alpha FT-IR equipped with a Pt-Diamond single-bounce ATR.

Elemental analysis was performed by the Microanalysis Laboratory at UIUC.

Single crystal X-ray data were collected at low temperature on either a Bruker D8 Venture DUO diffractometer with Photon III C14 detector and a Ag microfocus source (for [Cu­(II)­Me_6_Tren­(Cl)]­[Cl]) or a Bruker Kappa Apex-II DUO CCD diffractometer with Mo sealed tube source and Triumph curved graphite monochromator (for all others).

### Synthesis of Complexes

#### [Cu­(II)­Me_6_Tren­(NO_2_)]­[CF_3_SO_3_]

The complex was synthesized according to previously described procedures with modified purification procedures.^29^ Cu­(CF_3_SO_3_)_2_ (0.642 g, 1.77 mmol) and tris­(2-dimethylaminoethyl)­amine (Me_6_Tren) (0.402 g, 500 μL, 1.75 mmol) were dissolved in 60 mL of acetone. The light green solution turned to a dark blue color with solids remaining on the bottom upon addition of NaNO_2_ (0.245 g, 3.55 mmol). The suspension was stirred for 1 h at room temperature during which time the solid completely dissolved and the solution became dark green. The solvent was removed under vacuum and the remaining green solid was washed with 15 mL of isopropanol. An additional purification step involved dissolving the solid in acetonitrile, filtering the solution to remove impurities, and removing the solvent under vacuum. The solid was further dried under vacuum overnight to yield 638 mg of dark green powder (75%). ESI-MS (methanol): *m*/*z* found: 338.1740 which matches the literature value (*m*/*z* theoretical: 338.1617).^29^ MS was collected on a Bruker Amazon Speed ETD Ion Trap with an ESI. Confirmational IR frequencies (cm^–1^): 1449, 1366, 1331, 1292, 1265, 1224, 1199, 1174, 1148, 1065, 1030, 952, 939, 907, 807, 777.^29^ Anal. Calcd for CuC_13_H_30_N_5_O_5_F_3_S: C, 31.93; H, 6.18; N, 14.32. Found: C, 31.70; H, 6.11; N, 13.72. Dark green crystals suitable for X-ray analysis were obtained from slow solvent evaporation of a concentrated solution of the complex in methanol (CCDC 2513421).

#### [Cu­(II)­Me_6_Tren­(Cl)]­[Cl]

The complex was synthesized according to a previously described procedure.^33^ Me_6_Tren (0.415 g, 500 μL, 1.80 mmol) was added dropwise to CuCl_2_ (0.254 g, 1.89 mmol) to yield a light blue/green suspension. After the addition of 20 mL of dichloromethane, the solution was stirred for 1 h. Hexane (25 mL) was added to precipitate the product. The solution was filtered via vacuum filtration and the solid was dried overnight to yield 510 mg of a blue powder (78%). UV–vis (CH_3_CN): λ_max_ = 938 nm, ε_max_ = 447 M^–1^ cm^–1^.^33^ ESI-MS (CH_3_CN) *m*/*z* theoretical for [Cu­(II)­Me_6_Tren­(Cl)]^+^: 328.146; *m*/*z* found: 328.146. MS was collected on an Agilent 6230 TOF system coupled to an Agilent 1260 Infinity LC system in positive mode. Anal. Calcd for CuC_12_H_30_N_4_Cl_2_: C, 39.5; H, 8.29; N, 15.36. Found: C, 39.33; H, 8.23; N, 14.8. Light blue crystals suitable for X-ray analysis, obtained by slow evaporation from methanol, confirmed the structure of the product (CCDC 2513422).

#### [Cu­(II)­Me_6_Tren­(C_2_H_3_O_2_)]­[C_2_H_3_O_2_]·3H_2_O

This complex was synthesized similarly to the previous one. Me_6_Tren (0.216 g, 270 μL, 0.937 mmol) was added dropwise to Cu­(C_2_H_3_O_2_)_2_·H_2_O (0.182 g, 0.912 mmol). Addition of 20 mL of CH_2_Cl_2_ fully dissolved all solids and the solution was stirred at room temperature for 1 h. After removing the solvent under vacuum, the resulting blue oil was washed with pentane to produce a blue powder. The powder was dried under vacuum overnight (yield = 0.259 g, 61%). ESI-MS (methanol) *m*/*z* theoretical for [Cu­(II)­Me_6_Tren­(C_2_H_3_O_2_)]^+^: 352.19; *m*/*z* found: 352.19. MS was collected using a Waters Synapt XS ESI-Q-TOF MS system. Anal. Calcd for CuC_16_H_42_N_4_O_7_: C, 41.23; H, 9.08; N, 12.02. Found: C, 41.33; H, 8.78; N, 12.02. Slow diffusion of ether into a concentrated solution of the complex in CH_2_Cl_2_ yielded crystals suitable for single-crystal X-ray analysis (CCDC 2513423).

## Results and Discussion

### Electrocatalytic Nitrite Reduction

The complex [Cu­(II)­Me_6_Tren­(NO_2_)]­[CF_3_SO_3_], **Cu–NO**_**2**_ in Scheme 1, was synthesized and its electrochemical properties determined by cyclic voltammetry. An initial cyclic voltammogram (CV) of **Cu–NO**_**2**_ in an acetate buffer at pH 4.00, with KCl as a supporting electrolyte shows a quasi-reversible feature with an *E*_1/2_ of 0.16 V vs Ag/AgCl and a peak-to-peak separation of 96 mV (Figure 1, blue trace). This feature is attributed to the Cu^II^/Cu^I^ couple. Upon addition of 100 mM of NaNO_2_ the cathodic current increases, the return oxidation is no longer observed, and the voltammogram takes a characteristic catalytic S-shape (Figure 1, left, red trace). This suggests the complex promotes electrocatalytic nitrite reduction. During the collection of the voltammogram, in the presence of nitrite, bubbles were observed at the surface of the working electrode. As a control, the same solution in the absence of **Cu–NO**_**2**_ does not show any evidence of nitrite reduction in the potential window tested (Figure 1, left, gray trace). Direct nitrite reduction at the electrode surface is not observed until a much more negative onset potential of –0.50 V vs Ag/AgCl, with a peak potential of −0.73 V vs Ag/AgCl in these conditions (Figure S1). An initial controlled-potential electrolysis (CPE) was performed at an applied potential of −0.10 V vs Ag/AgCl with a reticulated vitreous carbon working electrode to assess the stability of the catalytic activity. A sustained reduction current was observed over time (Figure 1, right), coupled to the formation of bubbles on the working electrode (Figure S2).

**Figure 1:**
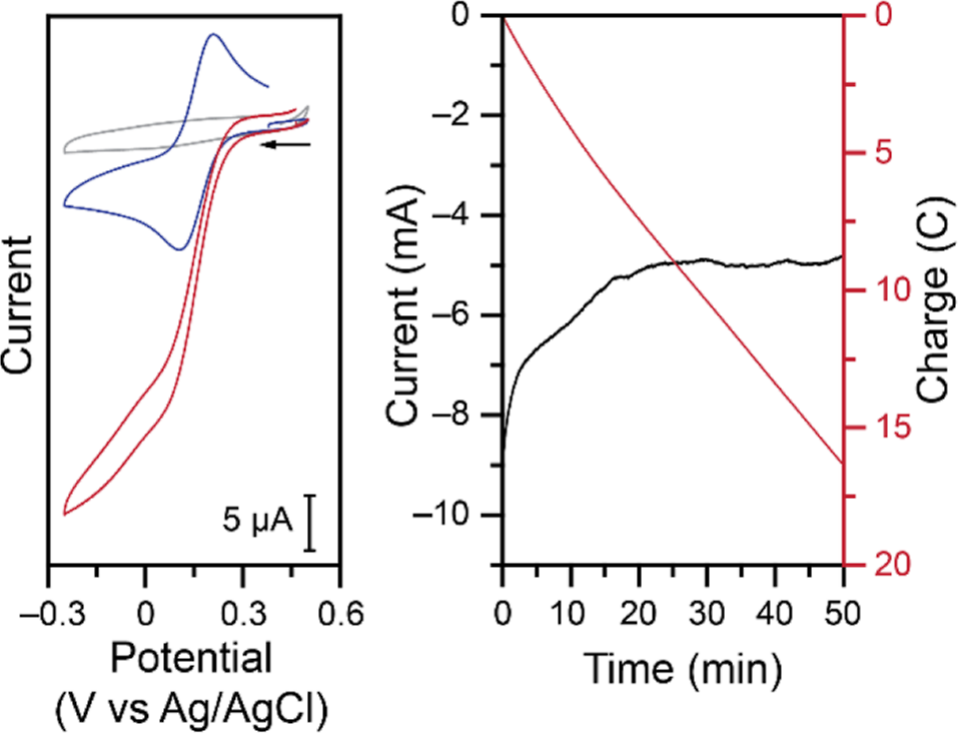
Left: CVs at 100 mV s^–1^ of 1.00 mM **Cu–NO**_**2**_ (blue trace), 100 mM NaNO_2_ (gray trace), and both mixed together (red trace) in 1.00 M KCl with 1.00 M acetate buffer at a pH of 4.00. Right: Current (black trace) and charge (red trace) vs time of a CPE at −0.10 V vs Ag/AgCl of 5.00 mM **Cu–NO**_**2**_ in 1.00 M pH 4.00 acetate buffer, 1.00 M KCl supporting electrolyte, and 100 mM NaNO_2_.

To identify the gases formed, a colorimetric test was performed: the gases evolved were collected and bubbled through a solution of cobalt tetraphenyl porphyrin (CoTPP) in CH_2_Cl_2_ following literature precedent for the colorimetric identification of NO_(g)_.^28,34,35^ The UV–vis spectroscopy spectra collected of the CoTPP solution show a redshift of the 531 nm absorbance band, characteristic of the binding of NO to the Co center (Figure S3). This confirms NO_(g)_ as the product. While the formation of N_2_O_(g)_ following further NO reduction has been reported in organic media,^29^ and for other Cu-based complexes,^36^ no evidence of electrocatalytic NO reduction by **Cu–NO**_**2**_ was observed when the initial solution containing the catalyst was sparged with 50 ppm of NO_(g)_ in N_2(g)_ (Figure S4). The following half-reactions for nitrite reduction were considered

1 $${{NO}_{2}}^{-}+1e^{-}+2H^{+}\to {NO}_{\left(g\right)}+H_{2}O$$

2 $$2{{NO}_{2}}^{-}+4e^{-}+6H^{+}\to N_{2}O_{\left(g\right)}+3H_{2}O$$

3 $$2{{NO}_{2}}^{-}+6e^{-}+8H^{+}\to N_{2\left(g\right)}+4H_{2}O$$

4 $${{NO}_{2}}^{-}+4e^{-}+5H^{+}\to {NH}_{2}{OH}_{\left(aq\right)}+H_{2}O$$

5 $${{NO}_{2}}^{-}+6e^{-}+8H^{+}\to {{NH}_{4}}_{\left(aq\right)}^{+}+2H_{2}O$$

The evolution of NO_(g)_ supports eq 1 as the operating pathway, following a simple 1-electron reduction of nitrite. This is confirmed by the observed change in nitrite concentration during CPE experiments. The concentration of nitrite was monitored using ion-exchange chromatography at the start and at the end of CPE experiments (Figure S5). These experiments were done using **Cu–Cl** as the catalyst to prevent the introduction of additional NO_2_^–^ into solution. These data confirm that the concentration of nitrite decreases over the course of the CPE, and that the change in nitrite concentration is consistent with a Faradaic process reducing 1 nitrite for every electron. The experiment was done in triplicate, yielding an average Faradaic efficiency (FE) of 99 ± 10% for the 1-electron electrocatalytic reduction of nitrite (Figure S5, Table S1). The near-quantitative Faradaic efficiencies for nitrite reduction imply a 1-electron reduction half-reaction. This would suggest NO_(g)_ as a product, which aligns with the observation of bubble formation on the working electrode during catalysis and the colorimetric test. Of note, nitrite was added to both the working and counter electrode compartments during the bulk electrolysis experiments. In the counter electrode compartment, the expected reaction is the 2-electron oxidation of nitrite, to generate nitrate. This is consistent with the observed increase in NO_3_^–^ and corresponding decrease in NO_2_^–^ concentrations at the counter electrode during CPE (Table S2).

To decipher the mechanism for the electrocatalytic reduction of nitrite by these copper complexes, further cyclic voltammetry studies were conducted. The nitrite-bound catalyst was used in these studies. CVs were first collected to determine the order in the NO_2_^–^ substrate (Figure 2). With additions of NO_2_^–^, a catalytic S-shaped curve develops. For a simple 1-electron EC’ catalytic reaction, the general form of the expected plateau current is given by the following equation^37^

6 $$i_{pl}=nFAC_{p}^{0}\sqrt{Dk_{obs}}$$

In this equation, *C*_p_^0^ is the concentration of the catalyst in the bulk, *n* is the number of electrons transferred to the catalyst from the electrode (here *n* = 1), *F* is Faraday’s constant, *A* is the area of the electrode, *D* is the diffusion coefficient of the catalyst, and *k*_obs_ is the observed rate constant. Analysis of the catalytic plateau current shows a linear relationship to the square root of the concentration of the substrate (Figure 2, right), which suggests that the reaction does follow the expected model in eq 6 and is first order with respect to substrate, with *k*_obs_ = *k*′[NO_2_^–^].

**Figure 2:**
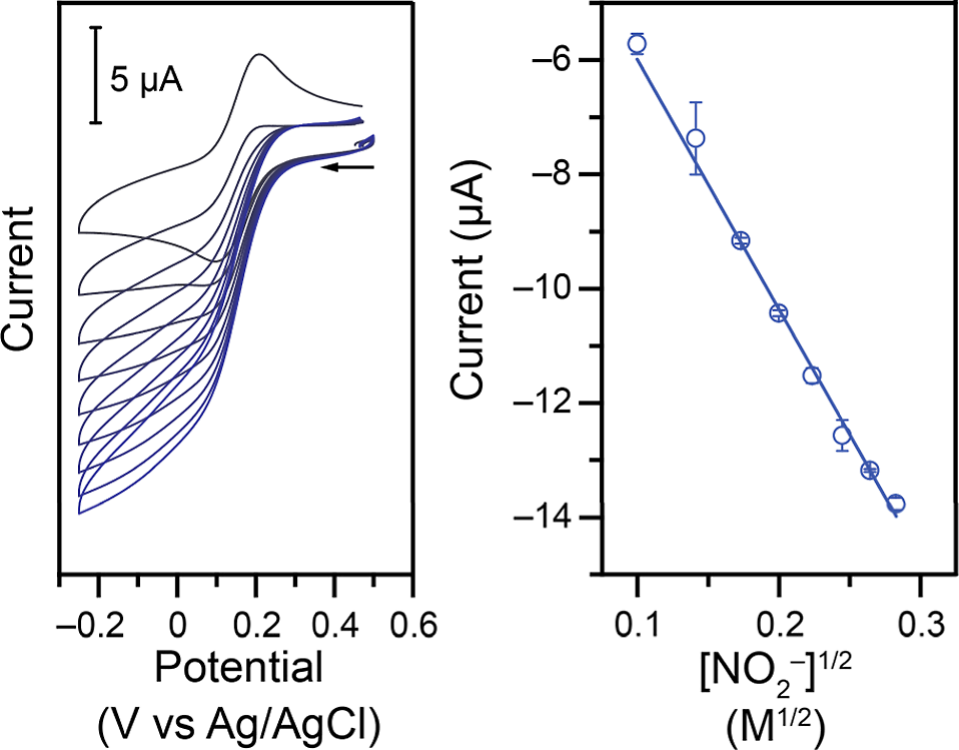
Left: Representative CVs at 100 mV s^–1^ of 0.50 mM **Cu–NO**_**2**_ in 1.00 M KCl electrolyte and 1.00 M acetate buffer at a pH of 4.00 upon addition of NaNO_2_ (nitrite additions from black to blue: 0, 10, 20, 30, 40, 50, 60, 70, 80 mM). Right: Corresponding analysis of the Faradaic current at 0.00 V vs Ag/AgCl (linear fit: *r* ^2^ = 0.998).

Next, the rate-order in the catalyst was determined. The catalyst **Cu–NO**_**2**_ was titrated into a solution containing 250 mM of NaNO_2_ and CVs were collected (Figure 3). The results show that, as expected, the current response is larger as more catalyst is added. The catalytic current was plotted versus the concentration of catalyst (Figure 3, right), and the linear fit confirms a rate-order of 1 with respect to the catalyst. This is again consistent with the catalytic plateau current in equation (eq 6).

**Figure 3:**
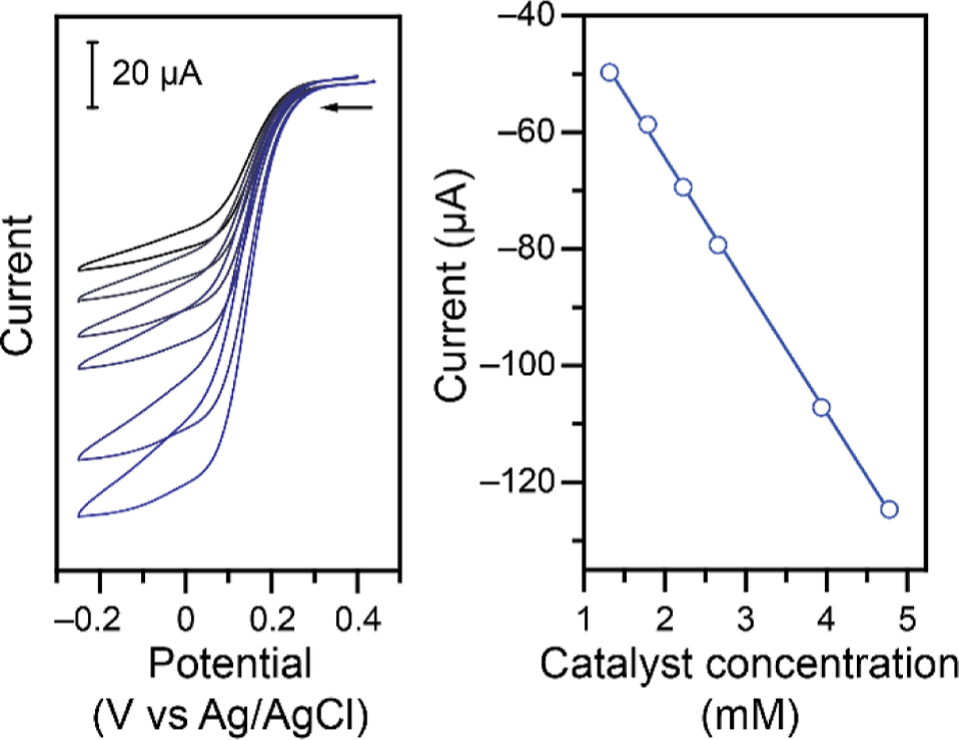
Left: CVs at 100 mV s^–1^ of 250 mM NaNO_2_ in 1.00 M KCl, 1.00 M acetate buffer at a pH of 4.00–4.06 in the presence of varying concentrations of **Cu–NO**_**2**_ (from black to blue: 1.3, 1.8, 2.2, 2.7, 3.9, and 4.8 mM). Right: Corresponding analysis of the Faradaic current at −0.10 V vs Ag/AgCl (linear fit *r* ^2^ = 0.999).

To determine if there is a rate-order difference between **Cu–NO**_**2**_ and **Cu–Cl**, the same experiment was repeated with a titration of **Cu–Cl**. Similarly, an apparent rate-order of 1 in catalyst is obtained (Figure S6).

Finally, following eq 1, a dependence in protons is also expected. When the pH of the buffer was systematically modified, several changes in the shape of the CVs are observed (Figure 4): (i) the current response gets larger the more acidic the solution, (ii) the onset potential shifts to more positive values as the pH is lowered, and (iii) the shape of the wave changes with pH, going from pseudoreversible to S-shaped with lowering pH values. This confirms pH plays a role in the reaction, but the role is more complex than expected (vide infra).

**Figure 4:**
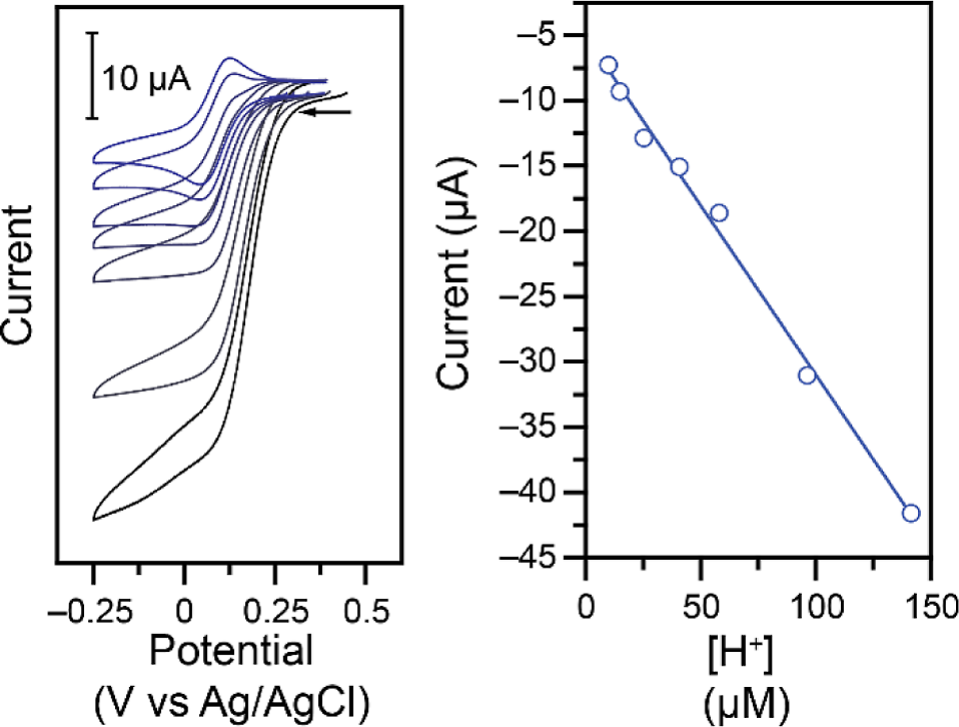
Impact of the pH of the buffer. Left: CVs at 100 mV s^–1^ of solutions containing 1.00 M KCl, 1.00 M acetate buffer, 1.00 mM **Cu–Cl**, and 100 mM NaNO_2_. The pH of the buffer was (from the black trace to the blue trace): 3.85, 4.02, 4.24, 4.39, 4.60, 4.83, 5.00. Right: Corresponding analysis of the Faradaic current at 0.00 V vs Ag/AgCl (linear fit: *r* ^2^ = 0.993).

On a first analysis, the evolution of the catalytic plateau current shows a linear evolution with the concentration of protons in solution, indicating an overall order of 2 in protons, consistent with eq 1. This would correspond to a general expression of the catalytic plateau current of the form

7 $$i_{pl}=FAC_{p}^{0}\sqrt{Dk^{\prime \prime }{\left[H^{+}\right]}^{2}\left[{{NO}_{2}}^{-}\right]}$$

While the catalysis seems straightforward, the system conceals complexity: the composition of the electrolyte and buffer has a significant and unexpected impact on the observation of catalytic activity at the probed potentials. While an estimate for the catalytic rate constant *k*″ could be calculated from Figure 4, it would rely on a value for the Faradaic peak current in the absence of substrate, and that value is not as straightforward as it would appear. The following section highlights this issue and demonstrates how electroanalytical techniques help tease out the underlying chemistry and rationalize the impact of the solution composition on catalytic activity.

### Identity of the Catalyst Resting State

While the catalyst as synthesized already has a bound nitrite ion, the electrochemical cell contains a number of other anions that could compete for binding to the Cu center. These include, in particular, Cl^–^ from the added KCl, and CH_3_COO^–^ from the buffer. All three Cu­(II) complexes considered have been independently synthesized and characterized by single-crystal X-ray crystallography (Figure 5).

**Figure 5:**
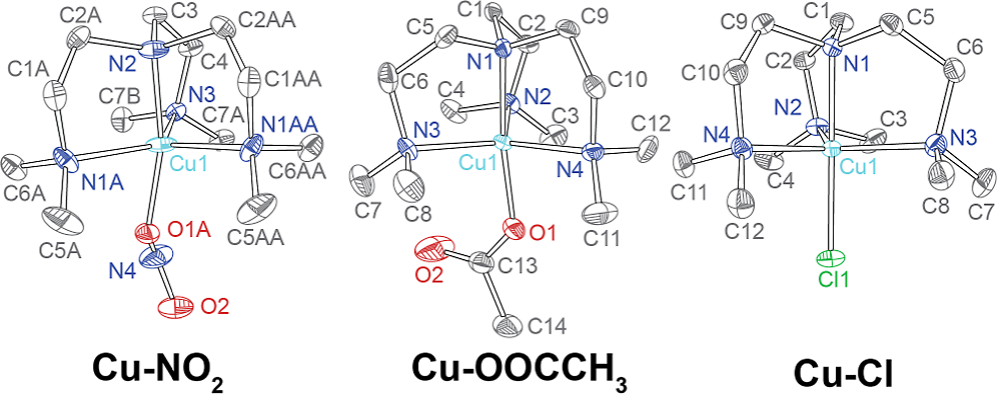
X-ray structures obtained for the 3 complexes independently synthesized [Cu­(II)­Me_6_Tren­(NO_2_)]­[CF_3_SO_3_], [Cu­(II)­Me_6_Tren­(C_2_H_3_O_2_)]­[C_2_H_3_O_2_]·3H_2_O, and [Cu­(II)­Me_6_Tren­(Cl)]­[Cl]. The ellipsoids are drawn at 50% probability. Hydrogen atoms, outer-sphere counteranions, and solvent molecules removed for clarity.

In all structures crystallized, the Cu­(II) is coordinated to the Me_6_Tren ligand in a tetradentate fashion. The remaining apical site is occupied by NO_2_^–^ (bound through an oxygen), by Cl^–^, or by CH_3_COO^–^. The structure of **Cu–NO**_**2**_ as synthesized with a triflate counterion matches the previously reported structure, though the structure here was refined in a higher symmetry space group compared to the previously reported structure.^29^ The other two structures are newly reported. Crystals grown from slow evaporation of water from a solution of **Cu–NO**_**2**_ in 1.00 M KCl were confirmed as [Cu­(Me_6_Tren)­Cl]­OTf, suggesting that the bound NO_2_^–^ is able to exchange with Cl^–^ in solution. Similarly, slow water evaporation from a solution of **Cu–NO**_**2**_ in an acetate buffer yielded crystals of [Cu­(Me_6_Tren)­OOCH]­OTf, which further emphasized the lability of the X^–^ ligand. More details on all those structures can be found in Figures S7–S11.

Given the established lability of the apical anionic ligand, and the possibility of differences between solution-state and solid-state behavior, further experiments were performed to better characterize the complex in solution. UV–vis spectroscopy was used to characterize solutions of the Cu­(II) complex in different conditions (Figure 6).

**Figure 6:**
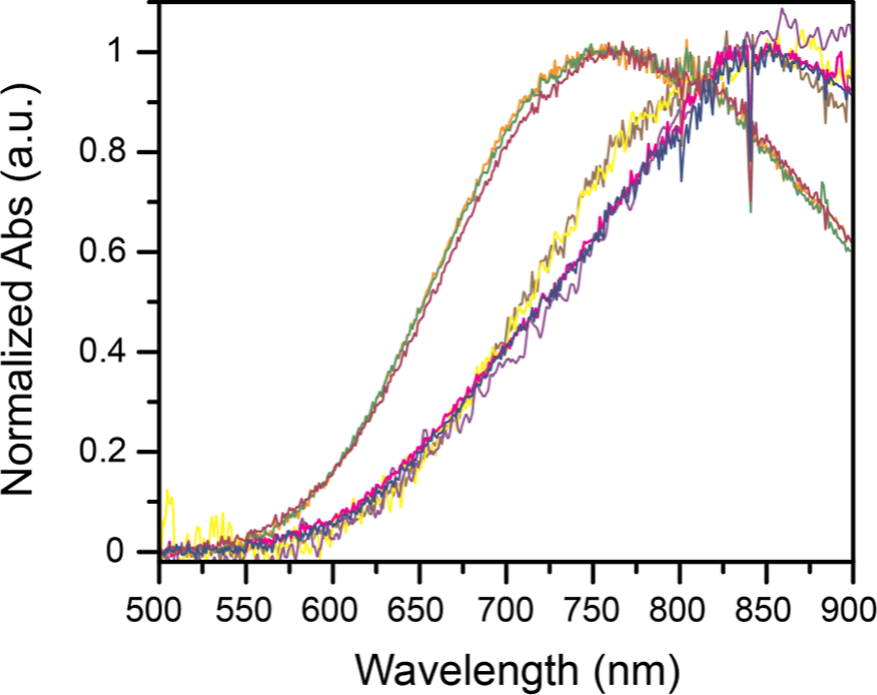
Normalized UV–vis spectroscopic data of 1 mM of **Cu–NO**_**2**_ (pink), **Cu–acetate** (purple), and **Cu–Cl** (blue) in water adjusted to pH 4.00 using HCl (for **Cu–Cl**), HNO_3_ (for **Cu–NO**_**2**_ and **Cu–acetate**), and KOH. A slight shift is observed when adding 1.00 M KCl (**Cu–Cl** yellow; **Cu–NO**_**2**_ brown). A large shift of the λ_max_ to 761 nm is observed when a 1.00 M pH 4.0 acetate buffer is used (**Cu–NO**_**2**_ orange; **Cu–Cl** dark red; **Cu–NO**_**2**_ + 1.00 M KCl green).

The UV–vis spectroscopic data (Figure 6) indicate that in the presence of acetate buffer, with or without added KCl, the absorbance feature shifts to 761 nm and is identical when starting with **Cu–NO**_**2**_, **Cu–acetate**, or **Cu–Cl**. This supports acetate binding to the Cu­(II) center in bulk solution in the presence of the acetate buffer. In the absence of the buffer, the feature shifts to 850 nm, regardless of the starting complex, which could point to H_2_O binding in the absence of the acetate buffer.

While the UV–vis spectroscopic data support an acetate ligand on the Cu­(II) center in solution at equilibrium, further experiments were needed to determine the resting state in electrochemical studies. To probe the influence of Cl^–^, a different electrolyte composition was sought for cyclic voltammetry studies. KNO_3_ was selected instead of KCl as NO_3_^–^ does not appear to replace NO_2_^–^ on the Cu­(II) center in solution (Figure S12). Indeed, crystals grown from a dilute solution of **Cu–NO**_**2**_ in the presence of 1 M KNO_3_ result in the same structure of the nitrite-bound complex crystallizing, with no exchange with nitrate observed.

Given these considerations, to first tease out the effect of acetate or chloride binding to the Cu center, a set of experiments was conducted using **Cu–NO**_**2**_ in a KNO_3_ electrolyte instead of KCl, and at a pH of 4, but in the absence of a buffer to avoid competition with the binding of acetate. In these conditions, at slow scan rates, the Cu­(II) to Cu­(I) reduction feature is irreversible, ill-defined, and a reoxidation is observed, which appears symmetric, consistent with a stripping peak suggesting plating of the reduced copper on the electrode surface (Figure S13, yellow trace). As the scan rate is increased, partial reversibility is restored and an *E*_1/2_ of −0.031 V vs Ag/AgCl is determined for the Cu­(II/I) couple in the absence of acetate or chloride. When Cl^–^ is added to the system, the pseudoreversible Cu­(II/I) wave is restored even at 100 mV s^–1^, and the *E*_1/2_ shifts to more positive potentials as more Cl^–^ is added (Figure 7).

**Figure 7:**
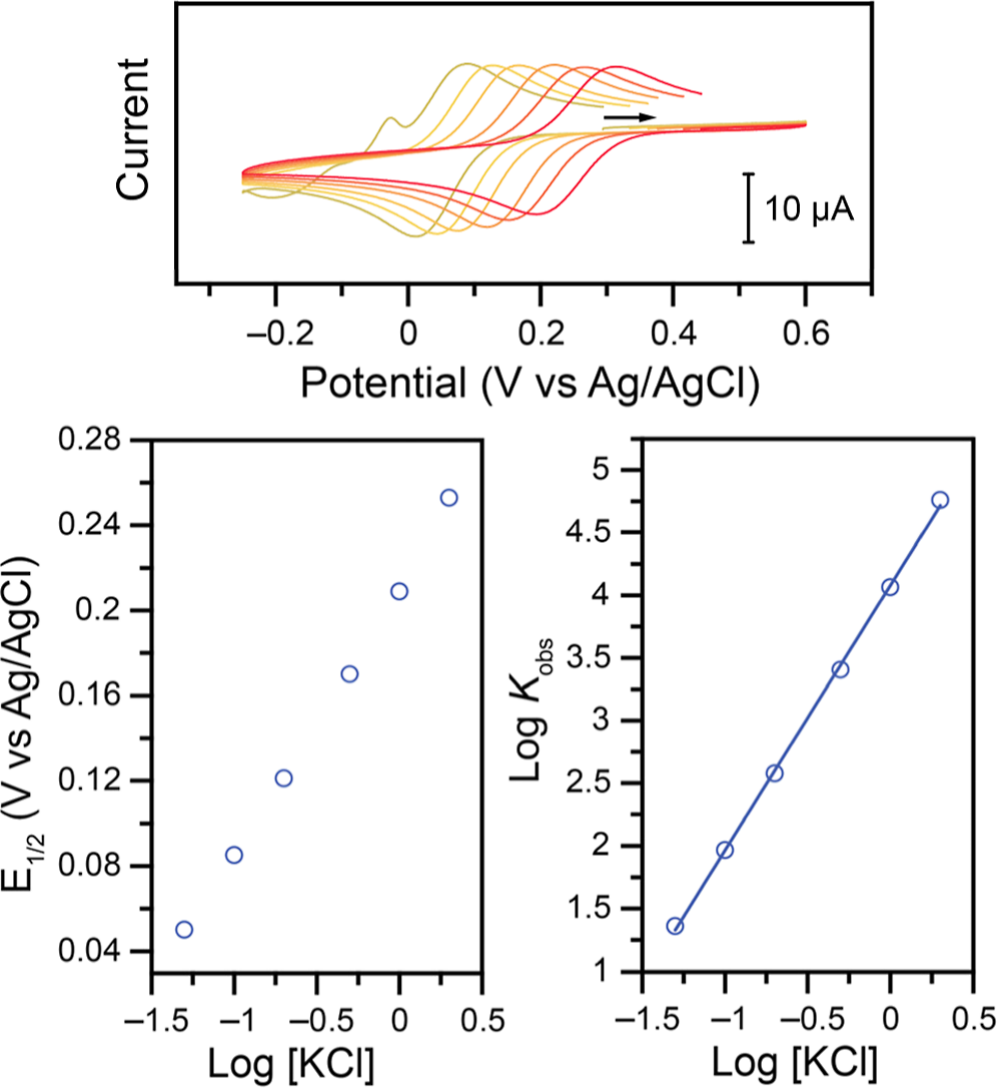
Top: CVs at 100 mV s^–1^ of 1.00 mM **Cu–NO**_**2**_ in 1.00 M KNO_3_ electrolyte, unbuffered, pH adjusted to 4.0 using HNO_3_ and KOH, after addition of (green to red) 0.05, 0.10, 0.20, 0.50, 1.00, and 2.00 M of KCl. Bottom: Resulting shift of the *E*_1/2_ (left) and log *K*_obs_ (right) as a function of log­[KCl]. The linear fit has a slope of 2.1 (*r* ^2^ = 0.999).

The evolution of the *E*_1/2_ for the Cu­(II/I) couple was measured as a function of the amount of KCl added to the system and is a clear indication of the involvement of Cl^–^ before or after the electron transfer (ET). Several possibilities were considered (Figure S14), but the data were only consistent with the following unexpected reaction

$$Cu\left(II\right)\mathrm{‐X}+e^{-}⇄Cu\left(I\right)\mathrm{‐X},E_{1/2}$$

$$Cu\left(I\right)\mathrm{‐X}+2{Cl}^{-}⇄{Cu\left(I\right)\mathrm{‐C}l}_{2},K_{eq}$$

In this framework, *E*_1/2_ is the value measured previously at pH 4 when starting from **Cu–NO**_**2**_ at fast scan rates in the absence of buffer and absence of KCl (*E*_1/2_ of −0.031 V vs Ag/AgCl, Figure S13). In this general mechanism, which does not keep track of charges, overall, the presumed X^–^ is lost and 2 Cl^–^ are added to the Cu center after reduction. This is surprising as anions are typically lost after the reduction of metal centers, however Cu­(I) centers are known to disfavor binding to nitrogen-rich ligands. As such, the possibility of nitrogen groups decoordinating from the reduced copper center becomes less surprising. This, in turn, would explain the stabilization of the reduced copper center by the chlorides. It should be noted that the analysis is agnostic to the identity, or existence, of X, which could be an open coordination site, or a solvent molecule, or possibly NO_2_^–^ (vide infra).

If the proposed mechanism above is followed, the equilibrium has an impact on the measured electrochemical step. This mechanism would lead to a specific shift of the observed redox potential as the change in the concentration of Cl^–^ influences the equilibrium and thus the concentration of Cu­(I)-X in solution. The equations underlying this mechanistic framework and examples of their adaptations to more complex equilibria have been reported,^38–40^ and more details are provided in the Supporting Information. In the EC mechanism, the observed equilibrium constant *K*_obs_ obtained would be of the form *K*_obs_ = *K*_eq_[KCl]^*n*^, where *n* is the stoichiometry of Cl^–^. As can be seen in Figure 7 (bottom right, slope of 2), the data support the involvement of 2 Cl^–^. Following this analysis, an equilibrium constant *K*_eq_ of 1.44 × 10^4^ M^–2^ is determined (Figure S14).

With a better understanding of the impact of Cl^–^, similar data sets were collected still at pH 4 but in the presence of an acetate buffer. As noted previously, as a stronger field ligand, acetate is expected to bind to the Cu­(II) center directly in the bulk solution. In a KNO_3_ electrolyte, still at pH 4 but with an acetate buffer, cyclic voltammograms obtained follow a similar trend to that observed in the absence of the buffer: the irreversible features regain reversibility and the *E*_1/2_ again shifts to more positive potentials in the presence of Cl^–^ (Figure 8). However, the *E*_1/2_ values are less positive than when the acetate buffer was not present. After 700 equiv of KCl were added (700 mM), the feature has an *E*_1/2_ of 0.140 V vs Ag/AgCl, compared to the value of 0.16 V vs Ag/AgCl observed in 1 M KCl electrolyte at the same pH as discussed above (Figure 1).

**Figure 8:**
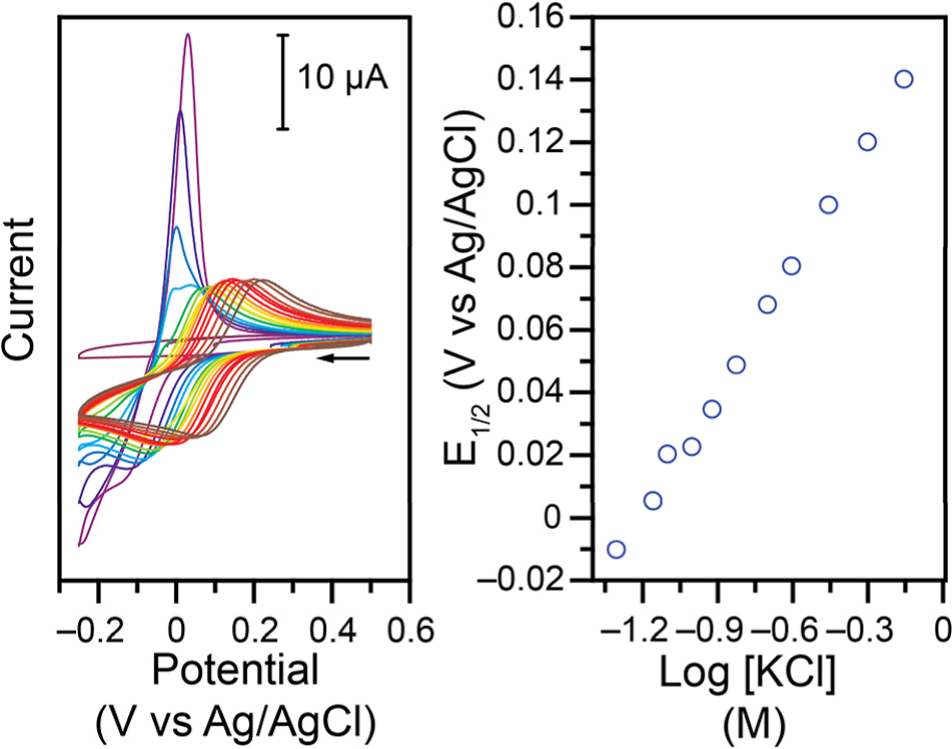
Left: CVs at 100 mV s^–1^ in 1.00 M KNO_3_ electrolyte, 1.00 M acetate buffer at pH 4.02–4.04, of (from purple to brown): the background, and of 1.00 mM **Cu–NO**_**2**_ in the presence of 0, 10, 20, 30, 50, 70, 80, 100, 120, 150, 200, 250, 350, 500, and 700 mM of KCl. Right: Corresponding evolution of the *E*_1/2_ on traces starting at 50 mM of KCl (linear fit *r* ^2^ = 0.996).

Similar analysis of the shift of the electrochemical feature as a function of the added KCl yields an observed equilibrium constant *K*_eq_^′^ of 1.61 × 10^3^ M^–2^, which would again correspond to an equilibrium constant for the addition of 2 Cl^–^ in these conditions with acetate present (Figure S15). This value is an order of magnitude smaller than in the absence of acetate buffer. This, coupled with the shift of the *E*_1/2_ to more positive values in the absence of acetate, supports the hypothesis of acetate binding to the Cu­(II) center in the bulk solution, as determined previously by the UV–vis spectroscopy study (Figure 6). The smaller value of the observed equilibrium constant calculated here would thus be explained by the requirement for acetate to decoordinate before reduction and Cl^–^ addition. Additionally, CVs collected at 100 mV s^–1^ using the **Cu–acetate** complex in an acetate buffer confirm that the reduction feature is irreversible in the absence of Cl^–^ but is pseudoreversible when KCl is used as the supporting electrolyte (Figure S16). The *E*_1/2_ of this wave is 0.16 V vs Ag/AgCl. This is the same *E*_1/2_ observed when starting with the **Cu–Cl** complex in similar conditions, once again highlighting the rich ligand exchange chemistry of this system (Figure S16).

Finally, a set of CVs were collected in which the pH is kept constant at a value of 4, and the electrolyte is a fixed 1 M KCl, but with varying buffer strengths from 0.1 to 1.0 M acetate buffer. In this set of experiments, reversible electrochemical features are obtained at all buffer strengths tested (Figure 9). Two trends are observed in the data: (i) the lower the buffer strength, the more positively shifted the *E*_1/2_, and (ii) the lower the buffer strength, the larger the peak currents. The first trend is consistent with the model proposed above, in which the decoordination of acetate is required to occur before the binding of Cl^–^. Indeed, the lower the buffer strength, with a fixed pH value, the lower the concentration of acetate, and thus the larger the ratio of Cl^–^ to acetate in solution. The second observed trend, that of the height of the feature changing as a function of buffer strength at a fixed pH, affords more information. The observation is consistent with a CE mechanism in which a chemical equilibrium occurs in solution, before the electron transfer. The extent of this equilibrium would control the concentration of the electroactive species, and thus the height of the redox wave. The following model can thus be proposed to explain the data

$$Cu\left(II\right)\mathrm{‐a}cetate⇄Cu\left(II\right)+acetate,K_{acetate}$$

$$Cu\left(II\right)+e^{-}⇄Cu\left(I\right),E_{1/2}$$

**Figure 9:**
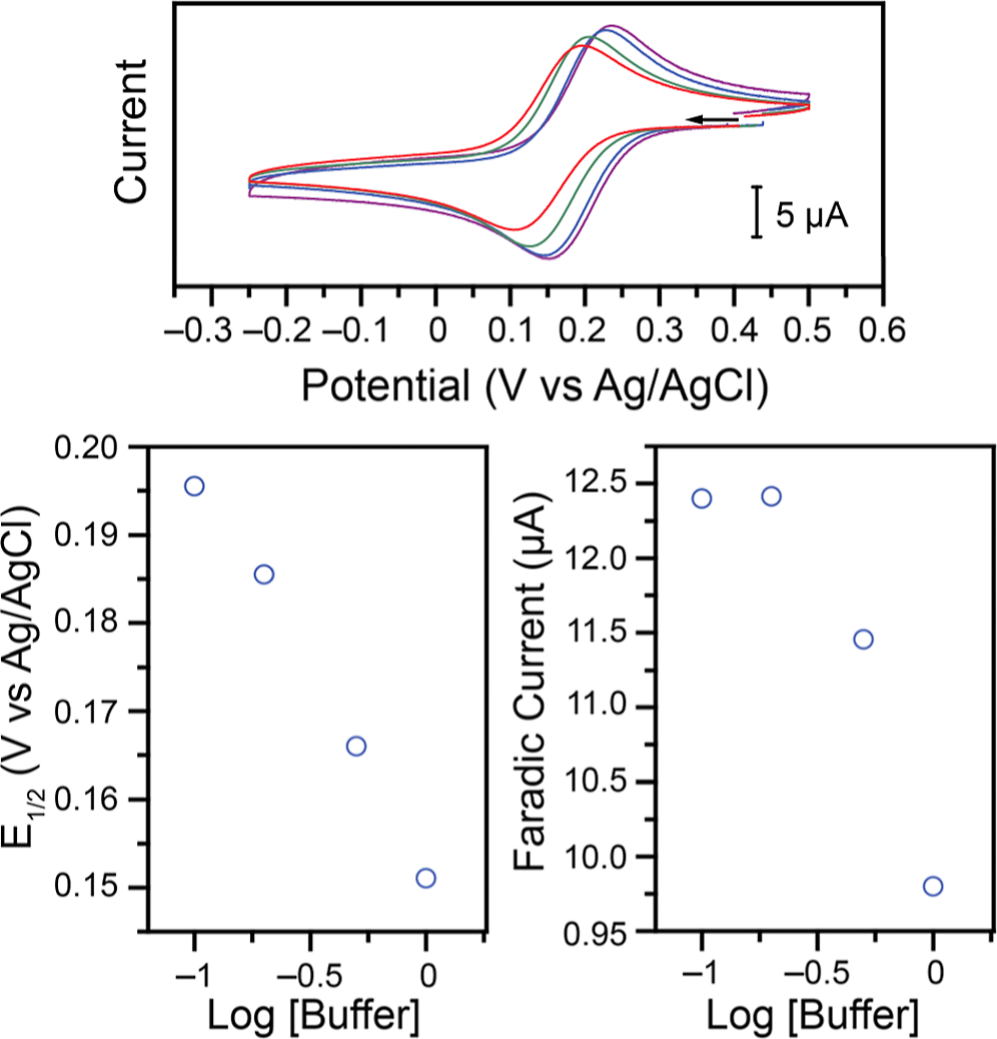
Top: CVs at 100 mV s^–1^ of 1.00 mM **Cu–NO**_**2**_, in 1.00 M KCl, with varying acetate buffer concentration at pH 4.0: 0.10 M (purple), 0.20 M (blue), 0.50 M (green), and 1.00 M (red). Evolution of the corresponding *E*_1/2_ (bottom left) and average Faradaic peak currents (bottom right).

This electron transfer step would then be followed by the chemical step previously identified

$$Cu\left(I\right)+2{Cl}^{-}⇄{Cu\left(I\right)\mathrm{‐C}l}_{2},K_{eq}$$

This is also supported by similar changes in peak heights and *E*_1/2_ values when comparing traces at 500 mM addition of KCl from the data sets in Figures 7 and 8 (without/with acetate buffer, see Figure S17).

### Revisiting the Influence of pH

To further characterize these electrochemical transformations in the absence of excess substrate, cyclic voltammetry experiments were repeated while varying the pH of the solution. The noninnocence of buffers has been increasingly noticed in recent years,^20,31,41–43^ and data above demonstrated that acetate is able to bind to the Cu­(II). Thus, CVs were collected at various pH values but in the absence of the acetate buffer. No clear shift in the *E*_1/2_ of the Cu­(II/I) feature was observed (Figure 10) when starting with **Cu–NO**_**2**_ (similar results when starting with **Cu–Cl** see Figure S18). Instead, the Cu­(II/I) feature grows at *E*_1/2_ = 0.209 V vs Ag/AgCl as the pH becomes more acidic, until reaching a maximum height at pH values lower than 4.5.

**Figure 10:**
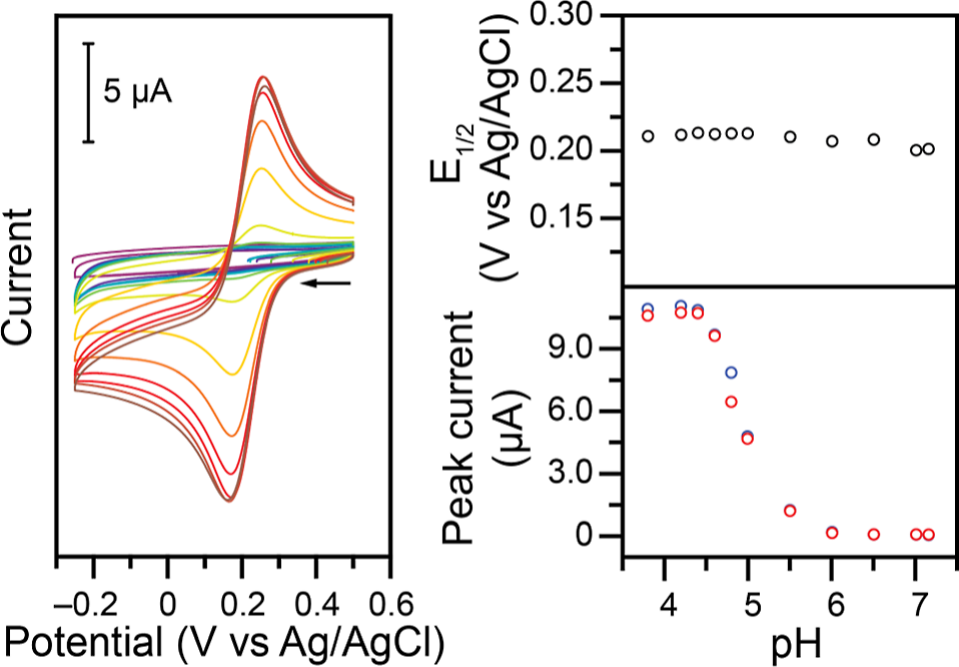
Left: CVs at 100 mV s^–1^ of 1.00 mM **Cu–NO**_**2**_ at varying pH in 1.00 M KCl, unbuffered. The pH, adjusted with HCl and KOH, was (from purple to red): 7.16, 7.01, 6.51, 6.01, 5.51, 5.00, 4.80, 4.60, 4.40, 4.20, and 3.80. Right: Resulting *E*_1/2_ vs pH (top) as well as cathodic (blue) and anodic (red) Faradaic peak currents vs pH (bottom).

The evolution of the shape of these voltammograms (Figure 10) is consistent with a CE mechanism in which an equilibrium chemical step (C) occurs in the bulk solution, before the electrochemical (E) reduction at the electrode. Based on the ability of the ligand to accept protons, the following steps are proposed

$$Cu\left(II\right)+H^{+}⇄HCu\left(II\right),K$$

$$HCu\left(II\right)+e^{-}⇄HCu\left(I\right),E_{1/2}$$

As discussed previously, Cl^–^ is then expected to bind to the Cu­(I) center, supplied by the 1 M KCl supporting electrolyte in this experiment.

By varying the pH and monitoring the height of the reversible Cu­(II/I) electrochemical feature, an estimate of the equilibrium constant for the chemical step can be determined. A value of *K* of 3.84 × 10^4^ is obtained, averaged across the anodic and cathodic measurements. From this value, an estimate of the p*K*_a_ of the Cu­(II) complex can be determined at 4.58 (Figure S19). The expected protonation site is one of the amino groups on the ligand. This is supported by work on tren-type ligands in the literature, in which the dissociation of one nitrogen from the metal center has been observed in mildly acidic conditions.^44–46^

While electroanalytical methods allowed to determine this p*K*_a_ value, the chemical step occurs in bulk solution at equilibrium. Therefore, a complementary spectrochemical titration experiment was performed to confirm this p*K*_a_ value. The evolution of the UV–vis spectroscopy absorption spectrum of the complex was monitored as a function of pH, in the absence of any buffers. The resulting data (Figure S20) yield an apparent p*K*_a_ value of 4.42, 4.43, and 4.48 when starting with **Cu–Cl**, **Cu–NO**_**2**_, and **Cu–acetate**, respectively. These numbers are in good agreement with the electrochemistry data discussed above (Figure S19).

With a better understanding of the effect of Cl^–^, of pH, and of acetate on the CVs obtained in the absence of the NO_2_^–^, the complexity of the data previously described in catalytic conditions at various pH levels is not surprising (Figure 4). These three factors contribute directly to the observed *E*_1/2_ of the Cu­(II/I) electrochemical feature, but also to the relative intensity of the observed feature. In the absence of acetate or Cl^–^, at pH 4, the reduction is observed at *E*_1/2_ = −0.031 V vs Ag/AgCl at fast scan rates. The presence of Cl^–^ shifts the wave to a value of +0.209 V vs Ag/AgCl. The presence of acetate modulates the magnitude of this shift, with a value of *E*_1/2_ = +0.16 V vs Ag/AgCl at 1 M KCl and 1 M pH 4 acetate buffer.

All three components are also critical for sustained electrocatalytic nitrite reduction by this complex. As the pH of the solution rises past 5.0, the catalytic currents are severely decreased (Figure 4), which can be explained by the identified p*K*_a_ for the complex. The presence of an acetate buffer is necessary for sustained catalysis to help maintain the pH as H^+^ are consumed during the electrocatalytic reduction of nitrite. Finally, the presence of Cl^–^ shifts the catalysis onset potential to much more positive potentials and, as such, diminishes the overpotential for catalysis. These last two effects were highlighted by collecting CVs at pH 4 in the presence or absence of Cl^–^ and of the acetate buffer (Figure S21).

### Proposed Catalytic Cycle

Overall, the catalytic cycle in Scheme 2 is proposed based on the observations described above and literature precedents.^28,31,34^ As discussed previously, the proposed chemistry is agnostic to the identity or existence of X which would be one of the anions in the solution or a solvent molecule. The overall charges of the complexes in Scheme 2 assume a monoanionic X. Since activity can be observed in the absence of the buffer or of Cl^–^, the scheme is proposed without these anions. Their impact will be discussed in greater detail below.

**Scheme 2:**
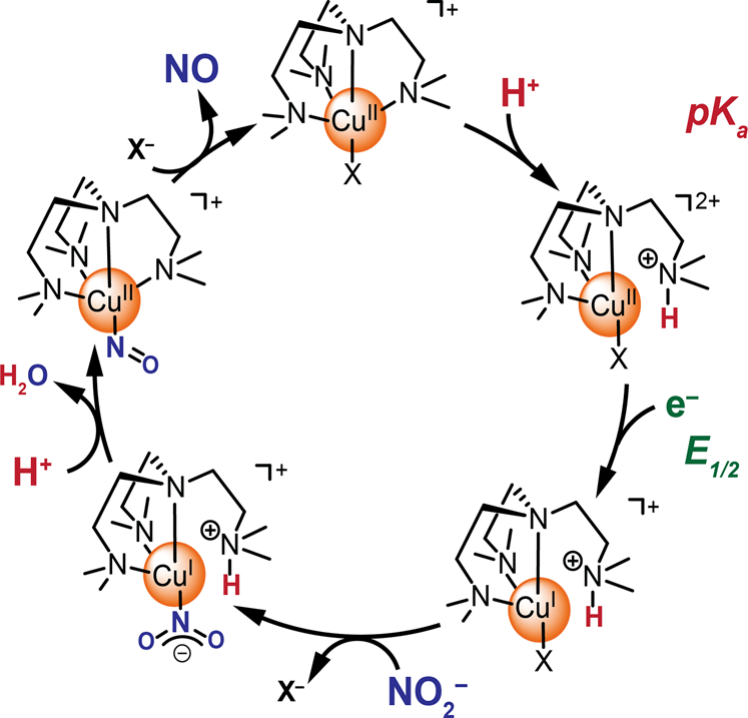
Proposed Catalytic Cycle in the Absence of Cl^–^

Starting with the Cu^II^ complex at the top of Scheme 2, a dimethyl amino arm of the ligand is protonated and decoordinates from the metal center. Evidence for this proton transfer was discussed with data in Figures S19, S20, and 10, and a p*K*_a_ value of 4.58 was determined through electrochemical methods. Since the electrocatalytic nitrite reduction data are collected at pH 4, this step is expected to be immediate once the Cu^II^ complex is dissolved in the solution. The next step is the electron transfer from Cu^II^ to Cu^I^, with an *E*_1/2_ of −0.031 V vs Ag/AgCl as determined from the variable scan rate data at pH 4 in the absence of acetate or Cl^–^ (Figure S13).

The following mechanistic steps show an overall order of 1 in the catalyst (Figure 3), an order of 2 in protons (Figure 4), an order of 1 in nitrite (Figure 2), and the production of 1 NO per electron in this process (Figures S2–S5, Table S1).

While the nitrite is bound to the Cu^II^ as a nitrito in the solid-state (Figure 5), there are several reports of a switch in the binding mode to a nitro once the copper center has been reduced from Cu^II^ to Cu^I^.^34,47^ The proposed cycle thus involves a nitro bound to the Cu^I^ center, followed by protonation of one of the oxygen atoms and release of a water molecule.^31,47^ The protonated amine on the ligand could be involved in shuttling a proton in this step, as has been reported for other ligands in organic media.^28^

The requirement of protons for the electrocatalytic reduction to proceed means a buffer would be necessary to sustain the activity of the catalyst. However, as has been observed in the literature, buffers can be noninnocent and even inhibit catalytic activity.^31,41,42,48–50^ A recent study reported that the nitrite electroreduction process is significantly complicated when a buffer is present.^31^ The work herein sheds light on some of these observations, in particular on the impact of Cl^–^ and acetate. In the presence of a pH 4 acetate buffer, the acetate conjugate base can bind to the Cu^II^ center (Figures 5 and 6). This leads to a negative shift in the half-wave potential of the Cu^II^/Cu^I^ couple (Figure 9), increasing the overpotential required for catalysis (Figure S21). Meanwhile, the presence of Cl^–^ plays a more unexpected role: while not required to observe catalytic activity (Figure S21), the presence of Cl^–^ stabilizes the Cu^I^ formed after the reduction. This is observed by the reversibility of the Cu^II^/Cu^I^ couple, which is regained in the presence of Cl^–^ (Figure S21). This means that during catalysis, if there is a mismatch between the mass transport of NO_2_^–^ to the electrode and the catalytic activity, the Cu^I^ catalyst could degrade or be overly reduced at the electrode surface, leading to stalled activity. In the presence of Cl^–^, however, the Cu^I^ center (Scheme 2, bottom right) could bind Cl^–^ (Figure 7), preventing deactivation of the catalyst. The presence of Cl^–^ also leads to a positive shift of the half-wave potential of the Cu^II^/Cu^I^ couple in the absence of acetate, from −0.031 to 0.209 V vs Ag/AgCl. In the context of electrocatalytic activity, this corresponds to a significant reduction of the overpotential by 240 mV.

## Conclusions

Electroanalytical methods were crucial in uncovering the unexpected chemistry underpinning the role of the buffer and other electrolyte components on the electrocatalytic activity of a simple metal complex. The underappreciated impact of these solution components should not be forgotten when screening complexes for electrochemical activity in water. For the specific case study in this report, the bioinspired copper-containing catalyst in a nitrogen-rich coordination environment is effective for the electrocatalytic reduction of nitrite in water. The hemilability of the ligand is proposed to facilitate the transfer of protons to the active Cu center. Meanwhile, the lability of the apical ligand trans to the central nitrogen on the Me_6_Tren ligand opens the way to modulating electrochemical activity through the presence of anions in solution. In particular, the presence of chlorides shifts the reduction potential to more positive values, diminishing the overpotential required for sustained electrocatalytic nitrite reduction. In contrast, the presence of acetate from the buffer has an opposite effect. The noninnocence of these ubiquitous components in electrochemical cells is crucial to consider when assessing catalytic activity. Ignoring these effects risks discontinuing work on promising catalysts.

## Supplementary Material



## Notes

Supporting X-ray characterization data, electrochemistry data, UV–vis spectroscopic data, and associated data analysis as referenced throughout the text (PDF)
