# Supplementary material for: Electroanalytical Methods to Establish the Role of Buffer and Electrolyte Components in Water Denitrification Using a Copper-Based Bioinspired Electrocatalyst
Source: ACS Meas Sci Au. 2026 Feb 10;6(2):507–16. doi: 10.1021/acsmeasuresciau.5c00203 (PMC13087953; doi:10.1021/acsmeasuresciau.5c00203)
Supplement: Supplementary file 1 [file tg5c00203_si_001.pdf]

Supporting Information

Electroanalytical Methods to Establish the Role of Buffer and Electrolyte Components in Water Denitrification Using a Copper-Based Bioinspired Electrocatalyst

*Vanessa A. Hulse, Katy A. Knecht, Frank R. Fronczek, and Noémie Elgrishi**

Department of Chemistry, Louisiana State University, Baton Rouge, Louisiana, 70803, United States

*Correspondence to noemie@lsu.edu

Index		*Page*
**Electrocatalytic nitrite reduction**		
CV of $NaNO_2$	Figure S1	SI-3
Picture showing gas generation during CPE	Figure S2	SI-3
Colorimetric test demonstrating NO formation	Figure S3	SI-4
CVs of the catalyst in the presence of NO	Figure S4	SI-4
Faradaic efficiency determination	Figure S5; Tables S1-S2	SI-5,6
CVs and analysis: order in catalyst using Cu-Cl	Figure S6	SI-7
**Identity of catalyst of the catalyst resting state**		
Single crystal X-Ray Crystallography data	Figures S7-S11	SI-8,9
UV-vis spectroscopic data with KNO_3	Figure S12	SI-10
CV data with KNO_3	Figure S13	SI-10
Analysis of CVs: KCl additions without acetate buffer	Figure S14	SI-12
Analysis of CVs: KCl additions with an acetate buffer	Figure S15	SI-13
CVs of Cu-acetate KNO_3 vs KCl	Figure S16	SI-14
Effect of acetate buffer on CVs of Cu-Cl with 0.5 M KCl	Figure S17	SI-14
**Revisiting the influence of pH**		
Effect of the pH on CVs of Cu-Cl	Figure S18	SI-15
Estimation of the pK_a based on CV data	Figure S19	SI-16
Estimation of the pK_a based on UV-Vis spectroscopy	Figure S20	SI-17
Effect of KCl and acetate on CV data	Figure S21	SI-18
**References**		SI-18

Electrocatalytic nitrite reduction

CV of $NaNO_2$

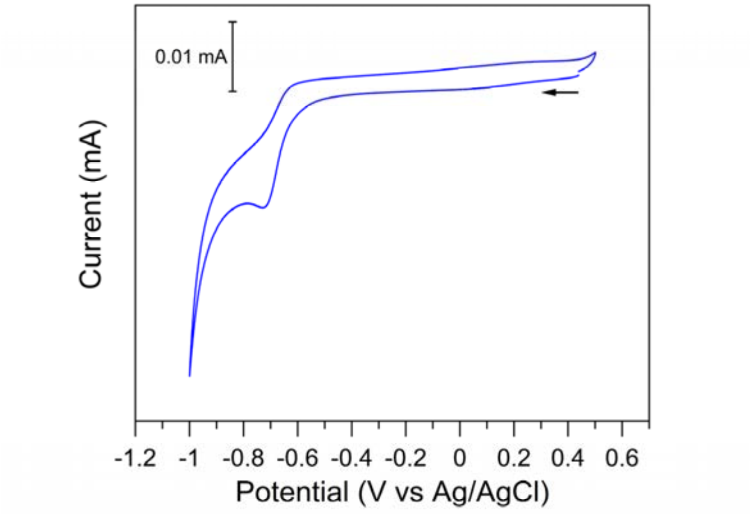

Figure S1. Control cyclic voltammogram of 100 mM $NaNO_2$ in 1.00 M KCl, 1.00 M acetate buffer at pH 4.0, at a scan rate of 0.100 V s^{-1}. Plotting convention: IUPAC.

Picture showing gas generation during CPE

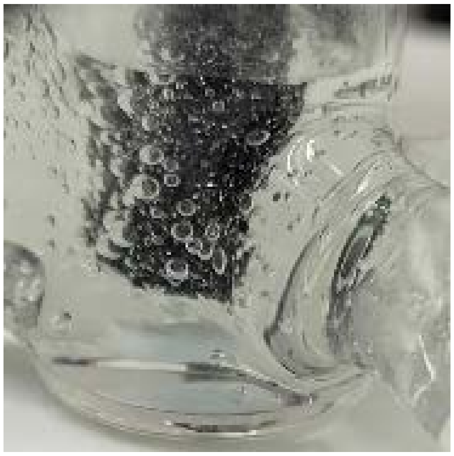

Figure S2. Picture of the working electrode compartment of the bulk electrolysis cell during a CPE at −0.10 V vs Ag/AgCl of 5.00 mM **Cu-NO$_2$** in 1.00 M pH 4.0 acetate buffer, 1.00 M KCl supporting electrolyte, and 100 mM $NaNO_2$. The picture shows the formation of bubbles at the surface of the Reticulated Vitreous Carbon (RVC) electrode.

Colorimetric test demonstrating NO formation

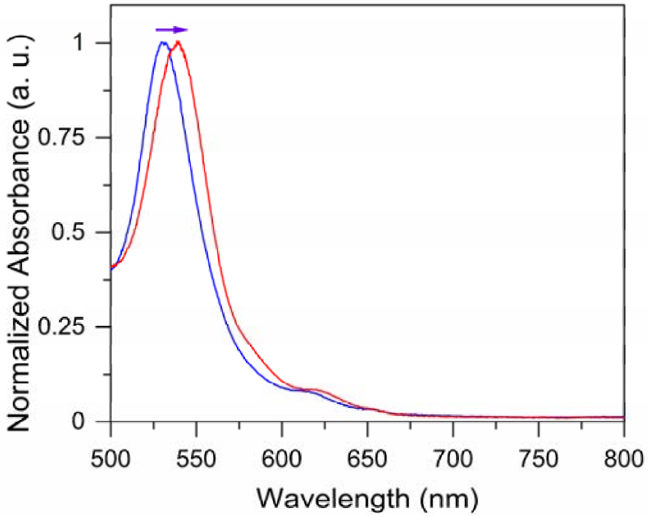

Figure S3. Normalized absorbance for a solution of 1.25 mM CoTPP in CH_2Cl_2 (blue trace) under N_2. A bulk electrolysis was set up with the following conditions: 1.00 M KCl, 1.00 M acetate buffer (pH 4.0), 153 mM $NaNO_2$, 5.3 mM Cu-NO_2. A fixed potential of −0.10 V vs Ag/AgCl was applied for two hours, during which time the headspace of the bulk electrolysis cell was continuously purged with N_2 and the outlet connected to a separate flask where the headspace was bubbled through the CoTPP solution. After the bulk electrolysis was stopped, the absorbance profile of the solution containing CoTPP was recorded again (red trace). The wavelength of the absorbance feature shifts from 531 to 539 nm, consistent with binding of NO to the cobalt center.[1–3]

CVs of the catalyst in the presence of NO

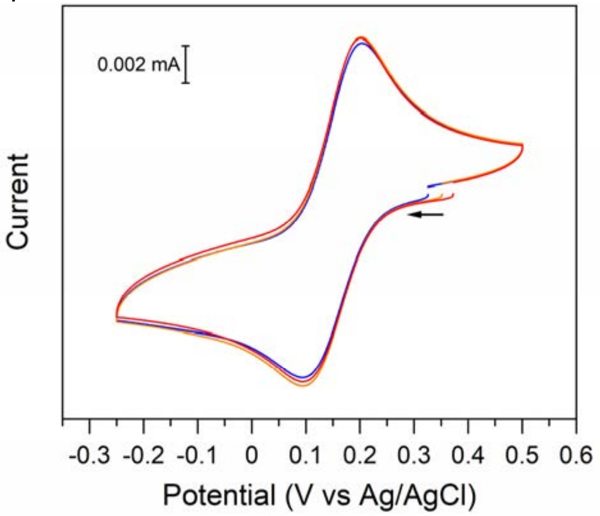

Figure S4. CVs of 1.00 mM Cu-NO_2 in 1.00 M KCl, 1.00 M acetate buffer (pH 4.1) under N_2 (blue trace), and after being sparged with 50 ppm NO in N_2 balance for 20 minutes (orange trace) and 60 minutes (red trace). Data collected at a scan rate of 0.100 V s^{-1}. Plotting convention: IUPAC.

Faradaic efficiency determination

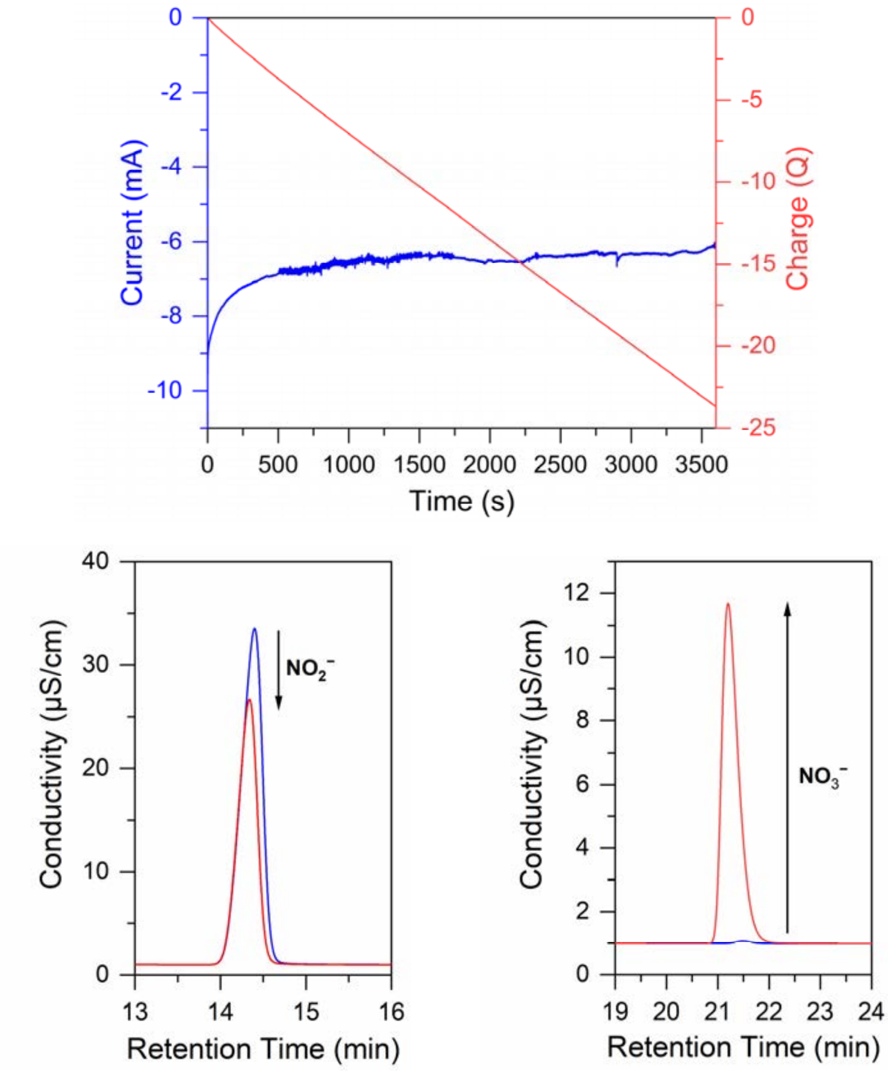

Figure S5. Top: Representative evolution of the current and charge during a CPE of 100 mM $NaNO_2$ with 5.00 mM **Cu-Cl** in 1.00 M acetate buffer (pH 4.0), 1.00 M KCl, at a fixed applied potential of −0.10 V vs Ag/AgCl. Data collected in a 2-compartment cell (10.0 mL working electrode and 5.00 mL counter electrode compartments) with an RVC working electrode. Bottom: Representative chromatograms before (blue) and after (red) CPE for the determination of nitrite in the working electrode compartment (left) and nitrate in the counter electrode compartment (right). Concentrations were determined using ion-exchange chromatography. The experiment was done in triplicate and data is presented in the tables below.

Table S1. Nitrite and nitrate concentrations in the 10.0 mL working electrode (**WE**) compartment during CPE (same conditions as in Figure S5).

	[**Cu-Cl**] (mM)	Time	$[NO_3^-]$ (mM)	$[NO_2^-]$ (mM)	$\Delta(NO_2^-)$ (mmol)	Charge Passed (C)	Faradaic Efficiency
**Trial 1**	5.00	Initial	2.87	98.3	−0.272	23.66	110.8%
		Final	3.12	71.1			
**Trial 2**	5.03	Initial	3.10	89.54	−0.231	23.45	94.9%
		Final	3.27	66.47			
**Trial 3**	5.02	Initial	2.73	87.36	−0.265	27.71	92.3%
		Final	2.82	60.84			
				**Average Faradaic Efficiency:**			**99 ±10%**

Faradaic efficiency (FE) was determined as the efficiency for reducing nitrite in the working electrode compartment through a 1-electron process:

$$FE = \frac{charge\ to\ reduce\ nitrite}{total\ charge\ passed\ during\ CPE} = \frac{\Delta(NO_2{}^-)\text{ in moles } \times\ F\ \times\ 1e^-}{total\ charge\ passed\ during\ CPE\ in\ C}$$

Table S2. Nitrite and nitrate concentrations in the counter electrode (**CE**) compartment during CPE (same conditions as in Figure S5).

	[**Cu-Cl**] (mM)	Time	$[NO_3^-]$ (mM)	$[NO_2^-]$ (mM)	$\Delta[NO_3^-]$ (mmol)	$\Delta[NO_2^-]$ (mmol)
**Trial 1**	5.00	Initial	2.61	98.02	+0.187	−0.189
		Final	40.0	60.27		
**Trial 2**	5.03	Initial	2.75	107.0	+0.181	−0.185
		Final	38.9	70.0		
**Trial 3**	5.02	Initial	2.73	106.9	+0.141	−0.163
		Final	30.97	74.25		

CVs and analysis: order in catalyst using Cu-Cl

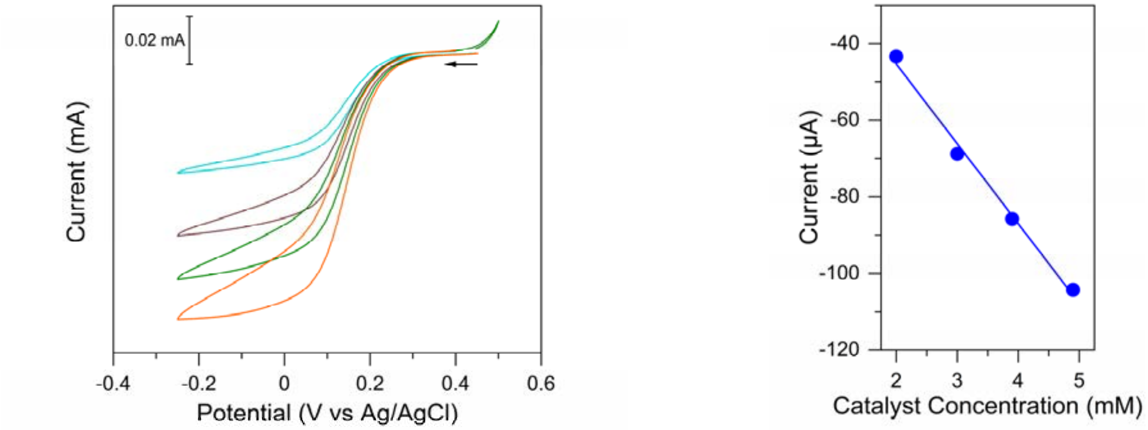

Figure S6. Left: CVs of the addition of 2.0 (blue), 3.0 (brown), 3.9 (green) and 4.9 (orange) mM of **Cu-Cl** into a solution of 250 mM of $NaNO_2$ in 1.00 M KCl, with a 1.00 M acetate buffer (pH 4.0-4.2). The data was collected on a 3 mm diameter glassy carbon electrode, at 0.100 V s^{-1}. Plotting convention: IUPAC. Right: corresponding plateau currents –0.10 V vs Ag/AgCl as a function of catalyst concentration showing a linear fit (r^2 = 0.990).

Identity of the catalyst resting state

Single crystal X-Ray Crystallography data

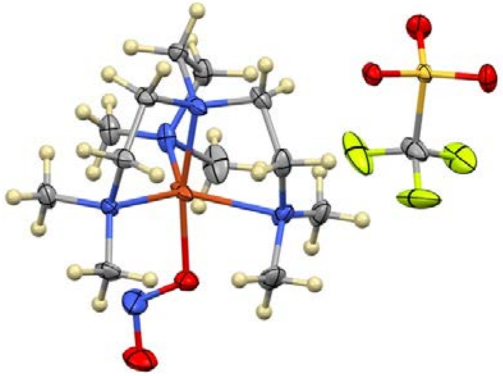

Figure S7. Crystallized by slow evaporation of a concentrated solution of the product from the synthesis of [Cu(II)Me_6Tren(NO_2)][CF_3SO_3] in methanol. Crystal structure deposited in the Cambridge Structural Database under CCDC 2513421.

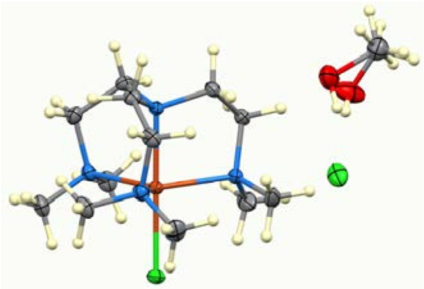

Figure S8. Crystallized by slow evaporation of a concentrated solution of the product from the synthesis of [Cu(II)Me_6Tren(Cl)][Cl] • MeOH in methanol. Crystal structure deposited in the Cambridge Structural Database under CCDC 2513422.

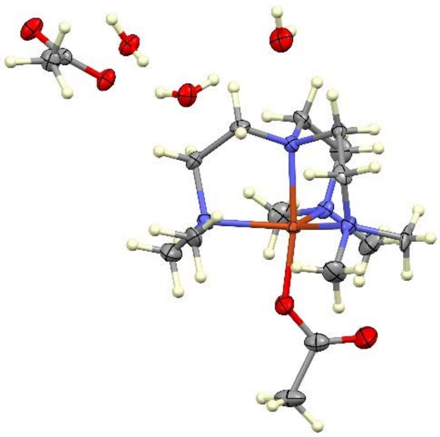

Figure S9. Crystallized by diffusion of Et_2O into a concentrated solution of the product from the synthesis of [Cu(II)Me_6Tren(CH_3COO)][CH_3COO]]•$3H_2O$ in CH_2Cl_2. Crystal structure deposited in the Cambridge Structural Database under CCDC 2513423.

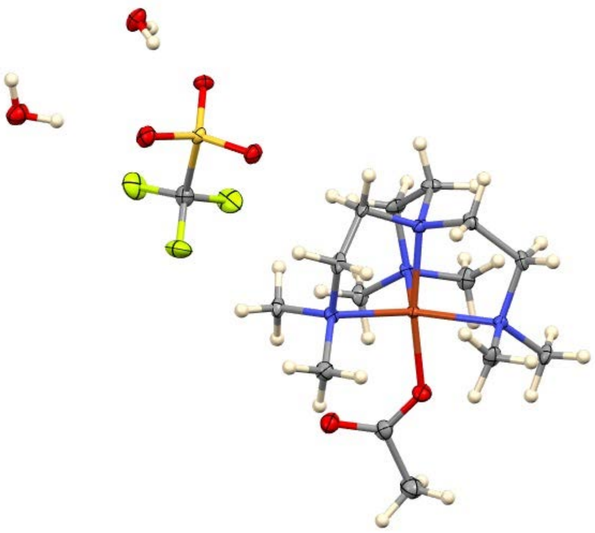

Figure S10. Crystallized by slow evaporation of water from a solution containing 82 mM of [Cu(II)Me_6Tren(NO_2)][CF_3SO_3] • $2H_2O$ in 0.5 M acetate buffer (pH 4.89). Crystal structure deposited in the Cambridge Structural Database under CCDC 2513424.

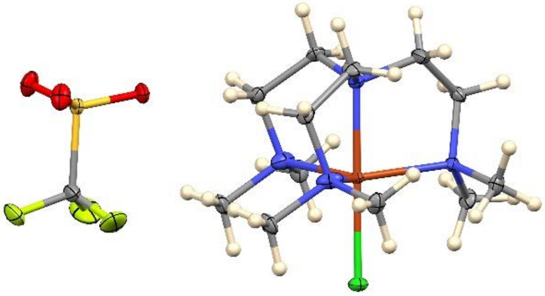

Figure S11. Crystalized by slow evaporation of water from a solution containing 72 mM of [Cu(II)Me_6Tren(NO_2)][CF_3SO_3] and ~3.5 M KCl. Crystal structure deposited in the Cambridge Structural Database under CCDC 2513425.

UV-vis spectroscopic data with KNO_3

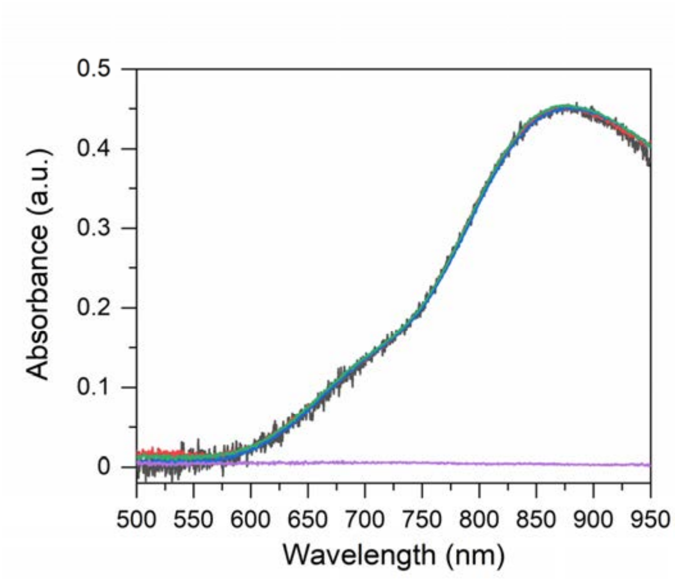

Figure S12. UV-vis spectroscopic data of 1 mM **Cu-NO$_2$** (red trace), **Cu-Cl** (blue trace) and 1.00 M KNO_3 (violet trace), as well as **Cu-NO$_2$** in the presence of 1.00 KNO_3 (black trace) and **Cu-Cl** in the presence of 1.00 M KNO_3 (green trace).

CV data with KNO_3

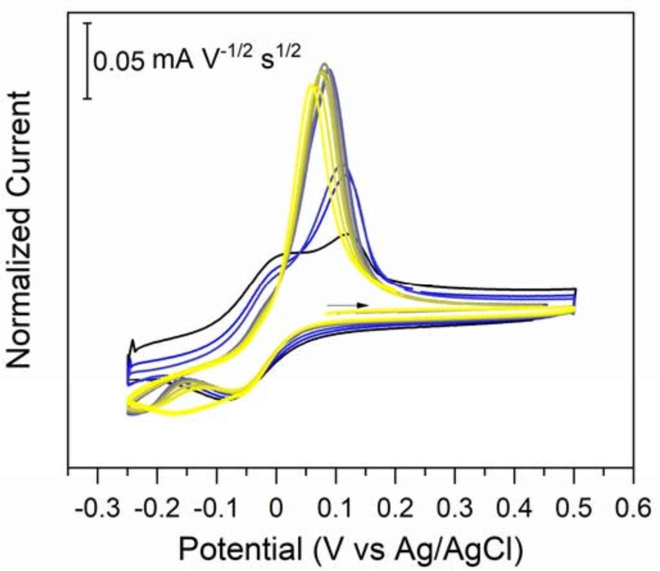

Figure S13. CVs of 1 mM **Cu-NO$_2$** in 1.00 M KNO_3, unbuffered, at a pH adjusted to 4.00 with KOH or HNO_3. The different traces correspond to varying scan rates, from (yellow to blue) 0.100, 0.200, 0.300, 0.400, 0.500, 0.750, 1.00, 5.00, 10.00 V s^{-1} and the final fastest scan rate at 20.00 V s^{-1} (black trace) in which partial reversibility is restored, with $E_{1/2}$ = –0.031 V vs Ag/AgCl.

Analysis of CVs: KCl additions without acetate buffer (Figure 7)

Several mechanisms were considered to analyze the effect of the presence of Cl^- on the Cu(II)/Cu(I) redox couple. The shapes of the voltammograms limit the possibilities: the Cu(II)/Cu(I) wave remains reversible, shifts with KCl addition, and retains its current magnitude. The mechanisms considered, presented below, are agnostic to the identity, or existence, of X^-, which could be, for example, an open coordination site, H_2O, or NO_2^-.

- CE mechanism: chemical step (C) first, followed by an electron transfer (E).

Given the shapes of the voltammograms, the possibility of a CE mechanism is limited to the zone DE of the standard zone diagram, in which a fully reversible wave is expected, at a $E_{1/2}$ which shifts based on the concentration of Cl^-.
Two possibilities are considered for the chemical step: associative or dissociative.

CE – Associative

First, a CE mechanism in which Cl^- binds before the electron transfer in an equilibrium step, following the simplified equation:

Cu(II)-X + Cl^- ⇄ Cu(II)-Cl + X^- K_{CEA}
Cu(II)-Cl + e^- ⇄ Cu(I)-Cl E^0

In this mechanism, the addition of KCl would generate the Cu(II)-Cl species, which would correspond to a reversible wave. The $E_{1/2}$ of the reversible wave would shift following the equation:[4]

$$E^{0\prime} = E^0 + \frac{RT}{F} ln \frac{K_{CEA}[Cl^-]}{1 + K_{CEA}[Cl^-]}$$

In this equation, $E^{0'}$ is the measured $E_{1/2}$ of the reversible wave that shifts with increasing amounts of KCl, E^0 is the $E_{1/2}$ of the reversible wave for the Cu(II)-Cl/Cu(I)-Cl redox couple, R is the gas constant (8.31447 J/mol K), F is Faraday's constant (96485.34 C/mol), T is the temperature (298 K), and *K* is the equilibrium constant. Rearranging the equation gives:

$$e^{\frac{(E^{0\prime}-E^0)F}{RT}} = \frac{K_{CEA}[Cl^-]}{1+K_{CEA}[Cl^-]} \Leftrightarrow \frac{[Cl^-]K_{CEA}}{e^{\frac{(E^{0\prime}-E^0)F}{RT}}} = 1 + K_{CEA}[Cl^-] \Leftrightarrow \frac{[Cl^-]}{e^{\frac{(E^{0\prime}-E^0)F}{RT}}} - [Cl^-] = \frac{1}{K_{CEA}}$$

This can be written as $z = \frac{1}{K_{CEA}}$ with $z = \frac{[Cl^-]}{e^{\frac{(E^{0\prime}-E^0)F}{RT}}} - [Cl^-]$. For every value of Cl^- added, $E^{0'}$ is measured, and a value of *z* can be calculated. If this mechanism is followed, the plot of *z* (=1/K) as a function of [Cl^-] would be expected to be constant. Using the value of $E_{1/2}$ recorded in the presence of the largest amount of KCl (the most positively shifted value) for E^0, the plot in Figure S14 (left) is obtained, which ***does not follow the mechanism*** as it would be expected to be constant.

CE – Dissociative

Next, a CE mechanism in which Cl^- is already bound at the start and leaves in an equilibrium step before the electron transfer, following the simplified equation:

Cu(II)-Cl ⇄ Cu(II) + Cl^- K_{CED}
Cu(II) + e^- ⇄ Cu(I) E^0

The more Cl^- is added to the solution, the harder reaching the Cu(II)/Cu(I) step is expected to be. *This does not match the data*, as addition of KCl (*i*) restored reversibility of the Cu(II)/Cu(I) wave, and (*ii*) shifts the wave to more positive potentials.

- EC mechanism: an electron transfer (E) followed by a chemical step (C).

Given the shapes of the voltammograms, the possibility of a EC mechanism is again limited to the zone DE of the standard zone diagram, in which a fully reversible wave is expected, at a $E_{1/2}$

that shifts based on the concentration of Cl^-. Similar to above, a mechanism in which Cl^- dissociates after a Cu(II)-Cl to Cu(I)-Cl reduction would not fit the data observed.

A scheme in which Cl^- is added after the electron transfer is considered below. This could occur in its simplest form as:

Cu(II)-X + e^- ⇄ Cu(I)-X $E_{1/2}$ (measured at fast scan rates, no Cl^-, no acetate)

Cu(I)-X + Cl^- ⇄ Cu(I)-Cl K_{EC1}

Following this model, the expected evolution of the $E_{1/2}$ as a function of the observed equilibrium constant K_{EC1} would be:[5] $E^{0\prime} = E^0 + \frac{RT}{F}\ln(1 + K_{EC1}[Cl^-])$

Where $E^{0'}$ is the measured $E_{1/2}$ of the reversible wave shifting with KCl, E^0 is the $E_{1/2}$ of the reversible wave in the absence of Cl^-, R, F, and T are defined as previously, and K_{EC1} is the equilibrium constant. E^0 is determined as –0.031 V vs Ag/AgCl from the data in Figure S13.

Using this equation, a value of K_{EC1} can be determined for each addition of KCl using:

$$K_{EC1}[Cl^-] = \left(e^{(E^0 - E^{0\prime})\left(\frac{F}{RT}\right)}\right) - 1$$

A plot of $\left(e^{(E^0 - E^{0\prime})\left(\frac{F}{RT}\right)}\right) - 1$ as a function of $[Cl^-]$ should be linear, with 0 as the y-intercept, if this mechanism is followed. The plot in Figure S14, middle, shows that *this mechanism is not followed*.

Analysis of the data obtained does not fit the CE mechanisms, and does not fit the simple EC presented above. However the data is consistent with the following unexpected reaction:

Cu(II)-X + e^- ⇄ Cu(I)-X $E_{1/2}$ (measured at fast scan rates; no acetate)

Cu(I)-X + 2 Cl^- ⇄ Cu(I)-Cl_2 K_{eq}

In which 2 Cl^- are added to the Cu center after reduction.

Following this model, the expected evolution of the $E_{1/2}$ as a function of the observed equilibrium constant K_{eq} would be:[5] $E^{0\prime} = E^0 + \frac{RT}{F}\ln(1 + K_{eq}[Cl^-]^2)$

Where the variables and constants are defined as above, with now K_{eq} as the equilibrium constant. E^0 is again determined as –0.031 V vs Ag/AgCl from the data in Figure S13 at the fastest scan rate, where partial reversibility is regained. Using this equation, a value of K_{eq} can be determined for each addition of KCl using: $K_{eq}[Cl^-]^2 = \left(e^{(E^0 - E^{0\prime})\left(\frac{F}{RT}\right)}\right) - 1$

A plot of $\left(e^{(E^0 - E^{0\prime})\left(\frac{F}{RT}\right)}\right) - 1$ as a function of $[Cl^-]^2$ should be linear, with 0 as the y-intercept, if this mechanism is followed, which is what is observed in Figure S14, right.

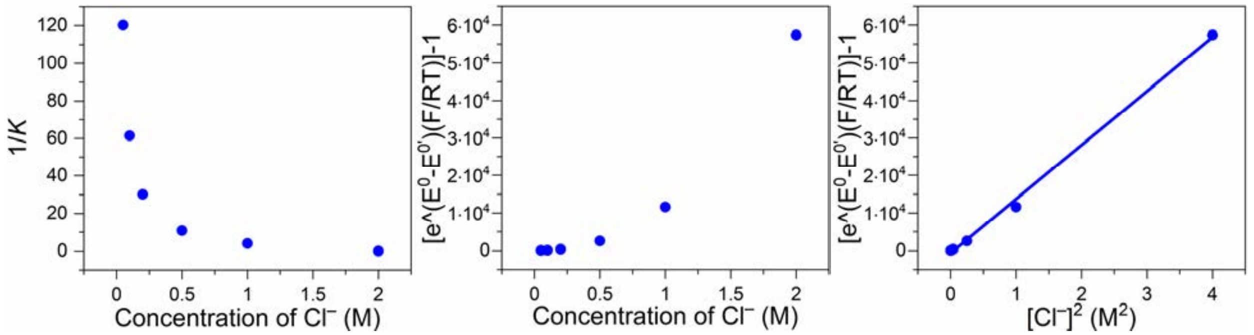

Figure S14. Analysis of the data in Figure 7 of the main text, assuming: (left) a CE associative mechanism, or an EC mechanism in which 1 Cl^- (middle) or 2 Cl^- (right) bind to the Cu center after reduction. Only the latter model fits the data: a linear fit with a slope of $K_{eq} = 1.44 \times 10^4$ M^{-2} ($r^2 = 0.997$) supports the proposed mechanism.

Analysis of CVs: KCl additions <u>with</u> an acetate buffer (Figure 8)

Similar to the analysis of the data in the absence of an acetate buffer, the data fit were obtained for the simple EC model in which the electron transfer occurs first, followed by an equilibrium addition of Cl^-. The model and underlying equation are:[5]

$Cu(II)\text{-}X + e^- \rightleftarrows Cu(I)\text{-}X$ $E_{1/2}$ (measured at fast scan rates; no acetate)

$Cu(I)\text{-}X + 2\,Cl^- \rightleftarrows Cu(I)\text{-}Cl_2$ K'_{eq}

$$E^{0\prime} = E^0 + \frac{RT}{F}\ln\left(1 + K'_{eq}[Cl^-]^2\right)$$

As described previously, if the data fits this model, a linear plot with 0 as the y-intercept would be expected in the following figure:

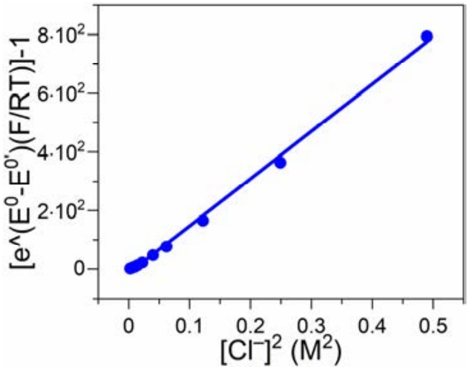

Figure S15. Analysis of the data in Figure 8 of the main text, assuming an EC mechanism in which 2 Cl^- bind to the Cu center after reduction. The plot is linear, with a slope of $K'_{eq} = 1.61 \times 10^3$ M^{-2} ($r^2 = 0.997$), which supports the proposed mechanism.

CVs of Cu-acetate KNO_3 vs KCl

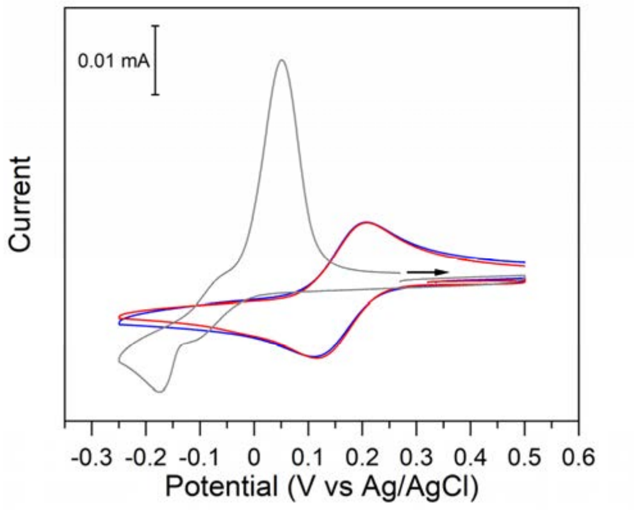

Figure S16. CVs of 1.00 mM **Cu-acetate** in a 1.00 M pH 4.0 acetate buffer adjusted with KOH or HNO_3, in the presence of 1.00 M KNO_3 (grey) or KCl (blue). The latter overlaps with a CV of 1.00 mM **Cu-Cl** in a 1.00 M pH 4.0 acetate buffer acetate with 1.00 M KCl (red, $E_{1/2}$ = 0.159 V vs Ag/AgCl). Data collected at 0.100 V s^{-1} and plotted in the IUPAC convention.

Effect of acetate buffer on CVs of Cu-Cl with 0.5 M KCl

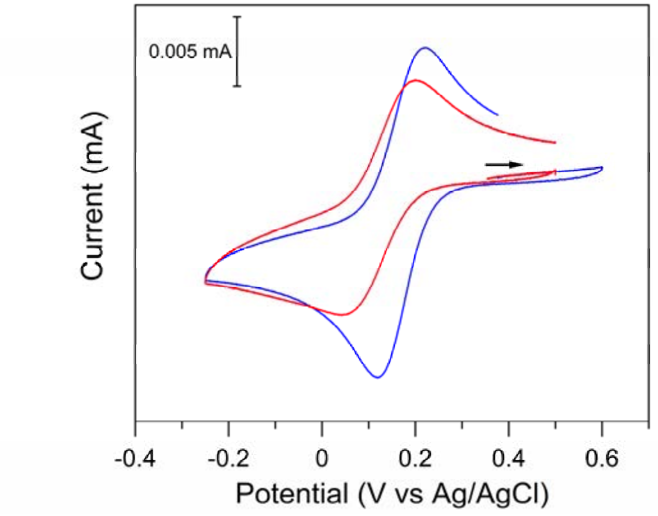

Figure S17. Comparison of CVs with (red trace) and without (blue trace) acetate buffer in solution. Both solutions contain 1 mM **Cu-Cl** in 1.00 M KNO_3 at pH 4.00 with 500 mM KCl. Data collected at 0.100 V s^{-1} and plotted in the IUPAC convention. Figure generated from data seen in Figures 7 and 8 in the main text.

Revisiting the influence of pH

Effect of the pH on CVs of Cu-Cl

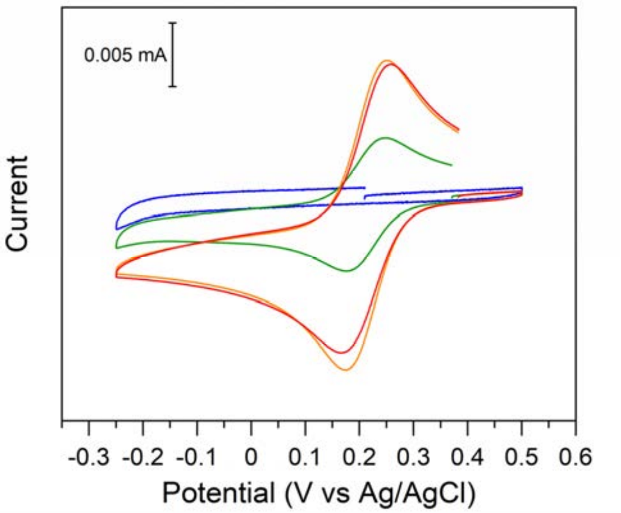

Figure S18. CVs of **Cu-Cl** at varying pH in 1.00 M KCl, unbuffered. The pH was adjusted with HCl and KOH to 6.8 (blue), 5.1 (green), 4.0 (orange), and 2.8 (red). Data collected at 0.100 V s^{-1} and plotted in the IUPAC convention.

Estimation of the pK_a based on CV data (Figure 10)

In Figure 10 of the main text, CVs are collected for 1.00 mM **Cu-NO$_2$** at varying pH in 1 M KCl, unbuffered. The values of the anodic and cathodic Faradaic peak currents (i_{pa} and i_{pc}) as a function of pH were extracted from these CVs. The shape of the voltammograms is consistent with a CE mechanism in the DO zone (diffusion zone)[5] where the equilibrium constant K is directly related to the peak current height. The following CE steps are proposed:

C: Cu(II) + H^+ ⇄ HCu(II) K

E: HCu(II) + e^- ⇄ HCu(I) $E_{1/2}$

Following this model, K_{eq} can be determined from the ratio of the peak currents measured (i_p) compared to the maximum peak currents (i_p^0) as a function of the concentration of H^+. This analysis was done for both the anodic and cathodic peak currents in the region in which the peak currents are changing, from pH 6.5 to pH 4.5.

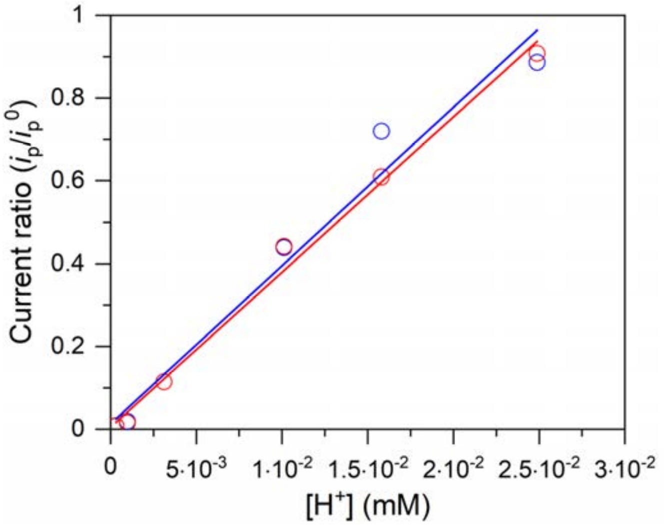

Figure S19. Cathodic (blue) and anodic (red) current ratios for the data in Figure 10 from pH 4.5 to pH 6.5. The linear fits have slopes of 37.5 ± 1.6 (r^2 = 0.99) and 38.2 ± 3.2 (r^2 = 0.99) for the anodic and cathodic data, respectively.

Converting the concentration of protons from mM to M gives an average value for K of 3.83 × 10^4. Recognizing that K corresponds to 1/K_a, this gives a value of 4.58 for the pK_a of the Cu(II) complex in these conditions.

Estimation of the pK_a based on UV-Vis spectroscopic data

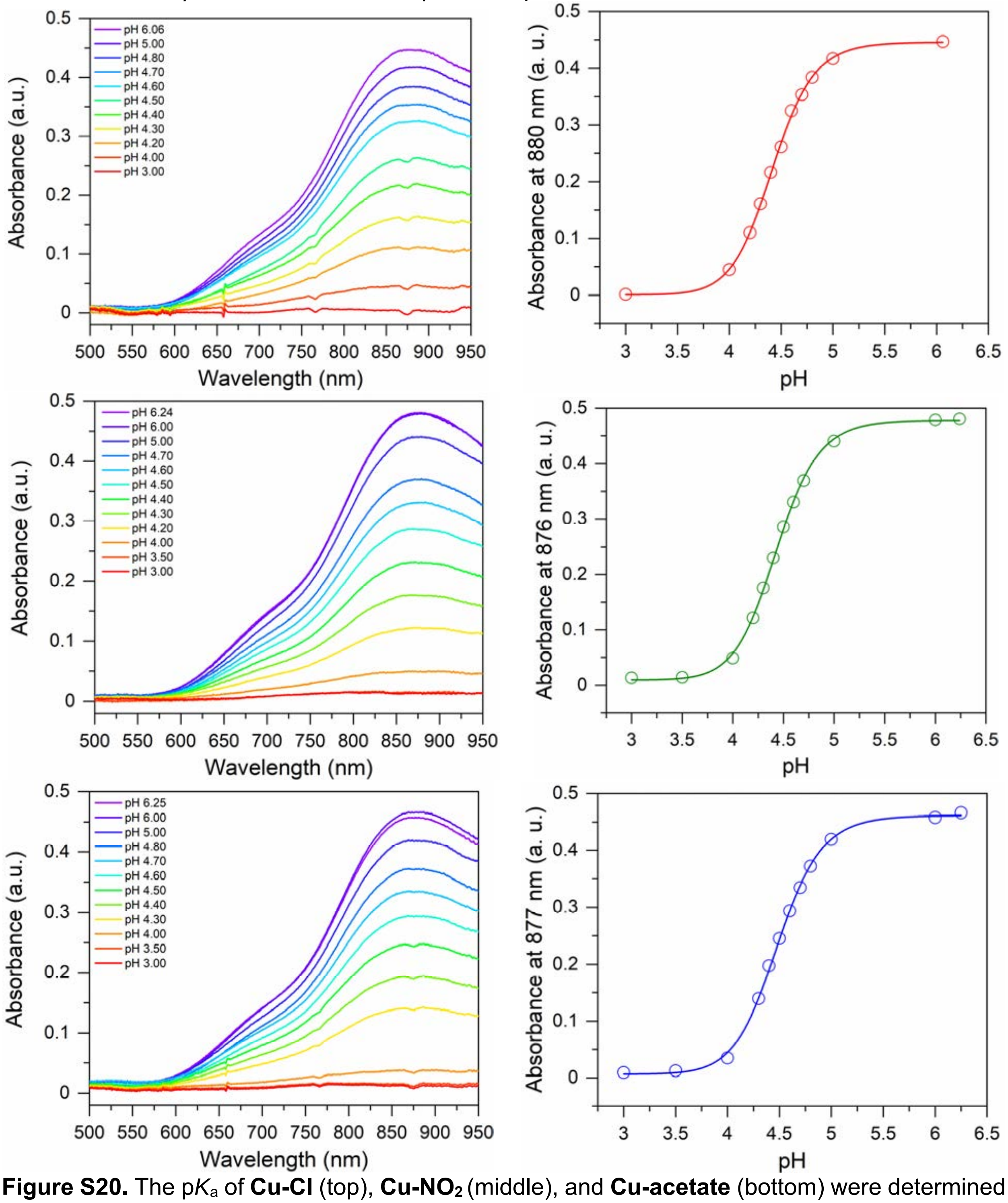

Figure S20. The pK_a of **Cu-Cl** (top), **Cu-NO$_2$** (middle), and **Cu-acetate** (bottom) were determined through spectrophotometric titrations. The absorbance of a 1.00 mM solution of each complex was measured as a function of the pH of the solution. The solutions were not buffered, no KCl was present, and the pH was adjusted with HNO_3 and/or KOH. A logarithmic fit of the absorbance at the λ_{max} as a function of pH (right) was used to determine a pK_a of 4.42 when starting with **Cu-Cl** (top, red, r^2 = 0.999), a pK_a of 4.43 when starting with **Cu-NO$_2$** (middle, green, r^2 = 0.999), and a pK_a of 4.48 when starting with **Cu-acetate** (bottom, blue, r^2 = 0.999).

Effect of KCl and acetate on CV data

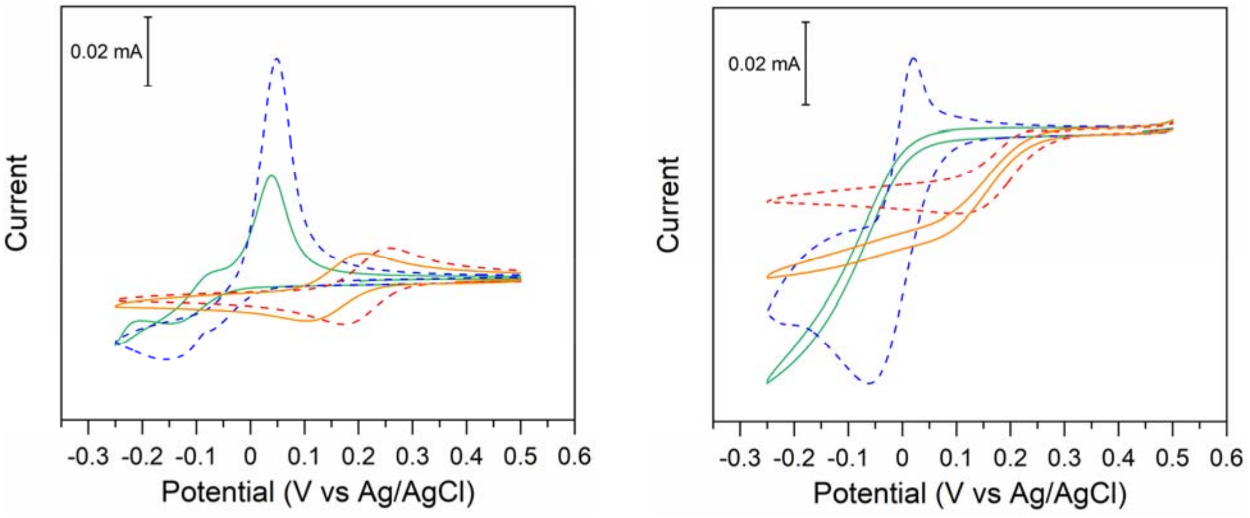

Figure S21. CVs of 1 mM **Cu-Cl** (green, blue, red) or **Cu-NO$_2$** (orange) in the absence (left) or presence (right) of 100 mM $NaNO_2$ and varying anions in solution. The electrolyte was 1.00 M of KCl (orange, red) or of KNO_3 (blue, green). The pH of all solutions was 4.0, either with 1.00 M acetate buffer (green, orange) or without a buffer (dashed traces, blue and red). The pH was adjusted to 4.0 with KOH and/or HNO_3 (blue, green) or HCl (orange, red). Data collected at 0.100 V s^{-1} and plotted in the IUPAC convention.

References

(1) Chandra Maji, R.; Mishra, S.; Bhandari, A.; Singh, R.; Olmstead, M. M.; Patra, A. K. A Copper(II) Nitrite That Exhibits Change of Nitrite Binding Mode and Formation of Copper(II) Nitrosyl Prior to Nitric Oxide Evolution. *Inorg. Chem.* **2018**, *57* (3), 1550–1561. https://doi.org/10.1021/acs.inorgchem.7b02897.

(2) Park, Y. J.; Peñas-Defrutos, M. N.; Drummond, M. J.; Gordon, Z.; Kelly, O. R.; Garvey, I. J.; Gullett, K. L.; García-Melchor, M.; Fout, A. R. Secondary Coordination Sphere Influences the Formation of Fe(III)-O or Fe(III)-OH in Nitrite Reduction: A Synthetic and Computational Study. *Inorg. Chem.* **2022**, *61* (21), 8182–8192. https://doi.org/10.1021/acs.inorgchem.2c00462.

(3) Yao, S. A.; Hansen, C. B.; Berry, J. F. A Convenient, High-Yielding, Chromatography-Free Method for the Insertion of Transition Metal Acetates into Porphyrins. *Polyhedron* **2013**, *58*, 2–6. https://doi.org/10.1016/j.poly.2012.05.038.

(4) Savéant, J. M.; Xu, F. First- and Second-Order Chemical-Electrochemical Mechanisms. *J. Electroanal. Chem. Interfacial Electrochem.* **1986**, *208* (2), 197–217. https://doi.org/10.1016/0022-0728(86)80535-6.

(5) Savéant, J.; Costentin, C. *Elements of Molecular and Biomolecular Electrochemistry*; Wiley, 2019. https://doi.org/10.1002/9781119292364.
